# Vorinostat impairs the cancer-driving potential of leukemia-secreted extracellular vesicles

**DOI:** 10.1186/s12967-025-06361-1

**Published:** 2025-04-10

**Authors:** Crescenzo Massaro, Giulia Sgueglia, Annamaria Muro, Damiana Pieragostino, Paola Lanuti, Maria Concetta Cufaro, Cristina Giorgio, Erika D’Agostino, Laura Della Torre, Serena Rubina Baglio, Marinella Pirozzi, Mariacarla De Simone, Lucia Altucci, Carmela Dell’Aversana

**Affiliations:** 1 Department of Precision Medicine, University of Campania “Luigi Vanvitelli”, 80138 Naples, Italy; 2 Department of Pathology, Cancer Center Amsterdam, Amsterdam UMC, Vrije Universiteit Amsterdam, 1081HV Amsterdam, The Netherlands; 3 Department of Innovative Technologies in Medicine & Dentistry, Center for Advanced Studies and Technology (CAST), University “G. d’Annunzio” of Chieti-Pescara, 66100 Chieti, Italy; 4 Department of Medicine and Aging Sciences, Center for Advanced Studies and Technology (CAST), University “G. d’Annunzio” of Chieti-Pescara, 66100 Chieti, Italy; 5 Research & Early Development (R&D), Dompé Farmaceutici S.p.A, Via De Amicis, 80131 Naples, Italy; 6 Istituto degli Endotipi in Oncologia, Metabolismo e Immunologia “G. Salvatore” (IEOMI)-National Research Council (CNR), 80131 Naples, Italy; 7 Stem Cell Transplantation Unit, Division Hematology, Cardarelli Hospital, 80131 Naples, Italy; 8 Department of Medicine and Surgery, LUM University, Casamassima, BA Italy; 9 BIOGEM, 83031 Ariano Irpino, Italy; 10 Medical Epigenetics Program, Vanvitelli Hospital, Naples, Italy

**Keywords:** Epigenetics, Extracellular vesicles, Leukemia, SAHA, Tumorigenicity

## Abstract

**Background:**

Leukemia-secreted extracellular vesicles (EVs) carry biologically active cargo that promotes cancer-supportive mechanisms, including aberrant proliferative signaling, immune escape, and drug resistance. However, how antineoplastic drugs affect EV secretion and cargo sorting remains underexplored.

**Methods:**

Leukemia-secreted extracellular vesicles (EVs) were isolated by Differential UltraCentrifugation, and their miRNome and proteomic profiling cargo were analyzed following treatment with SAHA (Vorinostat) in Acute Myeloid Leukemia (AML) and Chronic Myeloid Leukemia (CML). The epigenetic modulation of leukemia-secreted EVs content on interesting key target molecules was validated, and their differential functional impact on cellular viability, cell cycle progression, apoptosis, and tumorigenicity was assessed.

**Results:**

SAHA significantly alters the cargo of Leukemia-derived EVs, including miR-194-5p and its target BCLAF1 (mRNA and protein)*,* key regulators of Leukemia cell survival and differentiation. SAHA upregulates miR-194-5p expression while selective loading BCLAF1 into EVs, reducing the miRNA levels in the same compartment. Additionally, SAHA alters miRNA profile and proteomic composition associated with leukemic EVs, altering their tumor-supportive potential, with differential effects observed between AML and CML. Furthermore, in silico predictions suggest that these modified EVs may influence cell sensitivity to antineoplastic agents, suggesting a dual role for SAHA in impairing oncogenic signaling while enhancing therapeutic responsiveness.

**Conclusions:**

In conclusion, the capacity of SAHA to modulate secretion and molecular composition of Leukemia-secreted EVs, alongside its direct cytotoxic effects, underscores its potential in combination therapies aimed to overcoming refractory phenotype by targeting EV-mediated communication.

**Supplementary Information:**

The online version contains supplementary material available at 10.1186/s12967-025-06361-1.

## Introduction

Leukemia is characterized by extensive biological heterogeneity that often leads to relapse/refractory disease, making treatment extremely challenging [1]. In addition, the communication between cancer cells and surrounding stromal components plays a key role in different stages of the disease, further complicating the scenario [2]. Part of these interactions are mediated by extracellular vesicles (EVs), a heterogeneous population of vesicles secreted by all nucleated cells in the extracellular environment, previously regarded as a means to discard waste material [3, 4]. EV heterogeneity is the result of the different mechanisms and cellular compartments involved in vesicle biogenesis. According to current literature, EVs comprise: (i) exosomes (30–200 nm in diameter) originating in the endosomal compartment, where various mechanisms and pathways are involved, thus contributing to vesicle diversity [3, 4]; (ii) microvesicles (100–1000 nm in diameter) generated via direct outward budding of the plasma membrane [4]; (iii) apoptotic bodies (1000–5000 nm in diameter) deriving from apoptotic stimuli, which play a crucial role in maintaining tissue homeostasis and immune response yet their involvement in intercellular communication is generally considered less pronounced compared to exosome and microvesicles [3]. Therefore, we excluded such EV subtype from our study. Despite these classifications, the overlapping size range and the lack of reliable methods to distinguish between EV subtypes represent significant challenges [5, 6]. To address this, we adopted a size-based distinction between small vesicles (SVs) and large vesicles (LVs), acknowledging that vesicles of similar size (e.g. 150 nm) may include both exosomes and microvesicles. EVs contain various cargo molecules in both their lumen and membrane, including proteins, RNAs, miRNA, DNA, lipids, and metabolites, all of which can influence recipient cell phenotypes [7, 8]. As a result, accumulating evidence have highlighted the role of cancer-derived EVs in promoting tumor-supportive functions, including proliferation [9], angiogenesis [10], immune escape [11], and virtually every aspect of cancer biology [12]. Moreover, these vesicles are implicated in the development of refractory phenotypes in recipient cancer cells [13–17], further complicating treatment outcomes. Beyond investigating the mechanisms underlying this type of intercellular communication, growing efforts are focused on harnessing EVs for therapeutic applications. However, comparatively less attention has been paid to how therapeutics alter EV secretion and molecular composition, changes that can variably influence the phenotype and functionality of recipient cells.

In previous studies, we demonstrated that the imbalance between miR-194-5p and its target Bcl-2-associated transcription factor 1 (BCLAF1) drives the differentiation block and apoptosis resistance observed in Acute Myeloid Leukemia (AML) cell lines—hallmarks of cancer progression [18]. Treatment with SAHA (Vorinostat)—an FDA-approved HDAC inhibitor(NDC code: 0006–0568-40, CAS number: 149647-78-9, ATC code: L01XH01)—restored this imbalance, leading cancer cells toward apoptosis. Notably, SAHA not only downregulated BCLAF1 expression, at mRNA and protein level, but also affected its intracellular localization and function.

Here, we investigated whether SAHA modulates the cargo sorting of leukemia-derived EVs and examined the biological impact of these altered vesicles on surrounding tumor cells. We assessed SAHA-mediated effects in both acute myeloid leukemia (AML) and chronic myeloid leukemia (CML) [22]. Our study focused specifically on myeloid leukemias, given their poorer prognosis, lower survival rates, high relapse rates, and resistance to treatment compared to lymphoid leukemias [19, 20].

Additionally, previous studies have highlighted the significant role of soluble factors and EV secretion in the malignant progression of myeloid leukemia, particularly in the reprogramming of the immunosuppressive microenvironment [21]. In this context, EVs may represent a promising therapeutic target to disrupt tumor-supportive communication between leukemia cells and their microenvironment [19, 21].

Our aim was to elucidate the modulatory effects of SAHA on EV secretion and cargo composition in both AML and CML, overcoming the distinct genetic, epigenetic, and molecular backgrounds of these two myeloid leukemia models. Specifically, we sought to identify key leukemic processes and targets influenced by SAHA-induced EVs, proposing them as potential therapeutic candidates.

Our findings reveal that SAHA significantly alters EV secretion and content, thereby skewing their tumor-supportive potential in both AML and CML. Moreover, based on molecular observations and in silico predictions, such changes may enhance the sensitivity of residual Leukemia cells to specific therapeutic agents.

## Materials and methods

### Cell line culture and chemical compounds

Human cell lines used were U937 (AML; RRID: CVCL_0007)) and K562 (CML; RRID: CVCL_0004). Leukemia cell lines were grown in RPMI 1640 medium (EuroClone, Milan, Italy) supplemented with 10% heat-inactivated FBS (Sigma-Aldrich, St Louis, Missouri, USA) and 2% penicillin/streptomycin (EuroClone). EV-depleted FBS was used to culture cells for EV collection. Briefly, heat-inactivated FBS was ultracentrifuged at 100,000 × g for 12 h at 4 °C, without brake. The supernatant was added to 2% penicillin/streptomycin RPMI at a final concentration of 5%. SAHA (Vorinostat; Merck, Kenilworth, NJ, USA) was dissolved in DMSO (Sigma-Aldrich) and used at a final concentration of 5 μM. All human cell lines have been authenticated using STR profiling within the last 3 years. U937 and K562 cell lines were obtained from ATCC, Manassas, VA, USA. All experiments were performed with mycoplasma-free cells.

### Small and large vesicle isolation

Differential ultracentrifugation was performed using an Optima XPN-90 ultracentrifuge (Beckman Coulter, Brea, CA, USA) to isolate small and large vesicle subpopulations (SV and LV). A total of 4 × 105 leukemic cells/ml were plated in 30 ml RPMI containing EV-depleted FBS and treated with SAHA for 24 h before EV isolation. DMSO was added to the control (Ctr) groups. SVs were isolated as described previously [18]. The SV pellet was then carefully resuspended in 200 μl PBS for functional assays or 150 μl PBS for molecular analysis, and stored at − 80 °C. The same protocol was used to isolate EVs for vesicle characterization and miRNA profiling.

LV isolation was performed using the same steps as for SV isolation, but ending the protocol earlier, as LVs were pelleted at 10,000 × g. After centrifugation at 10,000 × g, the pellet was washed with PBS, collected, and referred to as LVs. LV preparations were resuspended in 200 μl PBS for functional assays or 150 μl PBS for molecular analysis, and stored at − 80 °C.

### EV sorting, staining, and acquisition

EVs were sorted and acquired as previously described [19]. Protocol details and variations are reported below. Reagent mix was prepared by adding 0.5 μl of fluorescein isothiocyanate (1)-conjugated phalloidin and lipophilic cationic dye (both from BD Biosciences, San Jose, CA, USA; #626267 custom) to 195 μl of PBS 1X. Rosetta Calibration (Exometry, Amsterdam, The Netherlands) was used according to the manufacturer’s instructions, to calibrate side scatter, relate side scatter in arbitrary units to standardized units of nm, as well as to the diameter and refractive index of particle. Samples were acquired by flow cytometry (FACSVerse, BD Biosciences). EVs were identified as LCD positive/phalloidin negative events. EV preparations of LCD positive/phalloidin negative events displayed distributions in the range of ~ 100–300 nm in diameter. Instrument performances were monitored by the Cytometer Setup and Tracking Module (BD Biosciences) and further validated by the acquisition of Rainbow Beads (BD Biosciences). The same settings were used for all other measurements. To stain EVs, 300 ml of CytoFix/CytoPerm 1X (BD Biosciences) was added to 200 ml of supernatants. After 10 min, 2 ml of anti-BCLAF1 was added. After 30 min of staining at room temperature (RT) in the dark, 200 μl of PBS 1X was added to each tube. Next, 1 ml of an anti-rabbit FITC-conjugated (Jackson ImmunoResearch, West Grove, PA, USA) was added to each tube and incubated for 30 min (RT, in the dark). Samples were acquired on a FACSVerse flow cytometer (BD Biosciences), as previously described [20, 21].

### Immunofluorescent staining and acquisition

Cells were plated at a confluence of 200,000 cell/ml in 2.5 ml medium using a 6-well plate. After 24 h incubation with SAHA (or DMSO as Ctr), 30,000 live cells were added to a 12-well plate containing round cover glasses (Thermo Fisher Scientific, Waltham, MA, USA; #10006111) pretreated with poly-L-lysine (0.1 mg/ml; Merck, #P4707) and centrifuged at 350 × g for 5 min. Cells were fixed with PBS 4% formaldehyde (Thermo Fisher Scientific, #PI28906) and incubated for 20 min at RT. Cell permeabilization was performed with PBS 0.1% Triton X-100 (Sigma-Aldrich, #9036-19-5) and incubated for 10 min at RT. Cells were blocked with PBS 10% serum (FBS) for 30 min at RT. Cells were incubated with the following primary antibodies diluted in PBS 1% BSA/PBS: 1:100 CD63 (BD Biosciences, #556019), 1:100 BCLAF1 (Thermo Fisher Scientific; #PA5-55686) and incubated for 1 h at RT. Cells were then incubated for 30 min with the following secondary antibodies: Goat anti-Mouse IgG conjugated with AlexaFluor 488 (Thermo Fisher Scientific; #A11029) 1:1000 and Goat anti-Rabbit IgG conjugated with AlexaFluor 594 (Thermo Fisher Scientific; #A11012) 1:1000, and incubated for 30 min. Lastly,round cover glasses were mounted on a microscope slide using one drop of mounting medium containing DAPI (Thermo Fisher Scientific, #P36935) for nuclei staining. Images were acquired using an SP8 LIGHNTING confocal microscope (Leica Microsystems, Wetzlar, Germany) using an S Plan Fluor ELWD 40X/0.60 objective (Nikon, Tokyo, Japan). Images were acquired with NIS-Elements software (Nikon).

### miRNA profiling

cDNA synthesis and real-time qPCR were performed using a miRCURY LNA Universal RT microRNA PCR system (Qiagen, Germany) according to the manufacturer’s instructions, as previously described [22]. Real-time PCRs were run on a 7900HT thermocycler (Applied Biosystems, Thermo Fisher Scientific) using thermal-cycling parameters recommended by Qiagen. Briefly, we adhered to the manufacturer’s protocol, where Raw Ct values were calculated using the RQ Manager software v.1.2.1 (Applied Biosystems), with manual adjustments for the threshold and baseline settings. Data were analyzed by applying a ΔRn threshold of 60 and subtracting the baseline between cycles 1–10. miRNA profiles were then determined using the delta-delta Ct method and EV-miRNA data were normalized on RNU6 levels.

### Target prediction analysis and GO enrichment

The prediction of target genes was performed using the miRSystem database as reported in [22], accomplished here by a value HIT ≥ 5, included validated genes, and observed/expected (O/E) ratio ≥ 2. For Gene Ontology (GO) enrichment and Kyoto Encyclopedia of Genes and Genomes (KEGG) pathway analysis was used the Database for Annotation, Visualization and Integrated Discovery (DAVID) (http://david.abcc.ncifcrf.gov/home.jsp). The Bonferroni Correction and the Benjamini–Hochberg Procedure were used in order to calculate the False Discovery Rate (FDR, p < 0.05) and the statistical significance at a p-value of < 0.05 was set.

### RNA isolation

Cells were collected by centrifugation at 350 × g for 5 min and lysed in 1 ml TRIzol (Invitrogen, Waltham, MS, USA). Similarly, 50 μl (out of 150 μl) of vesicle preparations were lysed in 1 ml TRIzol. RNA was extracted as previously described 9, with the addition of 3 μl glycogen (Roche) to the aqueous phase from vesicle preparations (but not to the cell lysate) before adding 500 μl of cold isopropylic alcohol, followed by vigorous shaking. RNA was resuspended in 10 μl DEPC-treated H_2_O (for vesicles) and 20 μl DEPC-treated H_2_O (for cells).

### Reverse transcription and real-time qPCR

mRNA was reverse transcribed using the SuperScript VILOTM cDNA Synthesis Kit (Invitrogen), according to the manufacturer’s instructions. A total of 1 μg RNA was reverse transcribed using a Peltier Thermal Cycler (MJ Research, Deltona, FL, USA). Gene expression was evaluated by real-time qPCR. A total of 50 ng of cDNA was amplified using the Power SYBR Green PCR Master Mix (Applied Biosystems, Waltham, MA, USA), according to the manufacturer’s instructions. Gene expression was normalized to GAPDH levels. Real-time qPCR was performed using a CFX96 detection system (Bio-Rad). The analysis was conducted using the ΔΔCt method. Primers used: BCLAF1 forward TCCGATCCATCTTTGACCACA, reverse BCLAF1 TGATACGAAGTGAACCGCTCG; GAPDH forward GGAGTCAACGGATTTGGTCGT, GAPDH reverse GCTTCCCGTTCTCAGCCTTGA.

### miRNA real-time PCR

After RNA extraction, the miRNA fraction was converted into cDNA and subsequentially miRNA Real-Time PCR was performed as described in [23].

### Western blotting

Cells and vesicles were lysed with RIPA buffer containing protease inhibitor cocktail (Roche, Switzerland), and the protein concentration was determined by BCA assay (Pierce, Etten-Leur, The Netherlands). Cell and vesicle lysates were diluted in different types of loading buffer according to the protein of interest. Blotting for vesicle-associated markers (CD63, CD81) required loading buffer without reducing agents, while blotting for other proteins was performed using loading buffer containing reducing buffer (beta-mercaptoethanol, Sigma- Aldrich, St. Louis, MO, USA), in both cases boiled at 95 °C for 5 min. Cell lysates and EV preparations in sample buffer were run on a 10% SDS gel and blotted on a nitrocellulose membrane (GE Healthcare, Eindhoven, The Netherlands). Membranes were incubated with antibodies against CD63 (BD Biosciences, San Jose, CA, USA; #556019) 1:300, BCLAF1 (Thermo Fisher Scientific, Waltham, MA, USA; #PA5-55686) 1:1000, and horseradish peroxidase-conjugated goat anti-mouse (Bio-Rad, Hercules, CA, USA; #1706516) 1:3 000 and goat anti-rabbit (Bio-Rad, #1706515) 1:3 000 antibodies.

### Transmission electron microscopy

EV pellets were fixed with 2.5% glutaraldehyde and all analysis were performed as described in [24].

### EV immunogold-labeling

Isolated EVs were loaded onto carbon-coated grids, fixed in 2% paraformaldehyde, washed, and then immune-labeled with anti-CD63 antibody (Abcam, United Kingdom; #ab59479), anti-CD9 antibody (Abcam, #ab92726), and anti-Tsg-101 (Abcam, #ab125011), followed by a 10 nm gold-labeled secondary antibody (Sigma-Aldrich). EVs were post-fixed in 2.5% glutaraldehyde, washed three times, contrasted with 2% uranyl acetate, and then examined with a JEOL 100CX transmission electron microscope (JEOL, Peabody, MA, USA).

### Cell viability assay

Cell viability was evaluated using a CCK8 kit (Sigma-Aldrich, #96992) following the manufacturer’s instructions. A total of 100 ul containing 10 000 cells/well were plated in a 96-well plate followed by the addition of 10 ul/well of WST-8 solution (1:10) and incubated overnight. The absorbance was then measured at 460 nm using an Infinite M200 plate reader (Tecan, Switzerland). Data were analyzed and presented as a percentage of live cells normalized to the relative control.

### Propidium iodide staining and cell cycle assessment

A total of 400,000/ml cells were transferred to polypropylene tubes, centrifuged at 350 × g, and the pellet was resuspended in 500 μl PBS. Propidium iodide (Sigma-Aldrich) was then added at a final concentration of 1 μg/ml, and cells were immediately analyzed by FACS Celesta Flow Cytometer (BD, Franklin Lakes, NJ, USA). For cell cycle assessment, cell cycle buffer was prepared by dissolving sodium citrate (10%; Sigma-Aldrich), NP-40 (10%; Sigma-Aldrich), and propidium iodide (2 mg/ml, Thermo Fisher Scientific) in PBS. Cells were resuspended in 500 μl cell cycle buffer, incubated for 15 min at room temperature, and then analyzed by FACS Celesta Flow Cytometer.

### Tumorigenicity assay

The tumorigenic capability of leukemic cells upon EVs w/wo SAHA treatment was evaluated by colony-forming unit assay using MethoCult (Stem cells, #SF H4236). A total of 500,000 leukemic cells/well were plated in a 6-well plate in 2.5 ml medium, to which 50 μl EV preparation were added, and incubated for 24 h. Subsequently, SAHA was added to the appropriate wells and incubated for 24 h. Following completion of the treatments, 10,000 cells were resuspended in 100 μl medium and added to 900 μl MethoCult medium. The mixture was then carefully pipetted to a new 6-well plate and incubated for five days. Images were acquired using a Cytation Cell Imaging Reader (Biotek, Winooski, VT, USA) at 4× magnification.

### Label-free proteomics analysis

To evaluate the protein cargo of sorted EVs from U937 medium, 700,000 purified EVs were used for proteomics analysis by comparing EVs of the two cell lines treated with SAHA to related controls. Samples were prepared for the Filter Aided Sample Preparation(FASP) protocol. EVs were lysed by sonication on ice (U200S sonicator control, IKA, Staufen, Germany) at 70% amplitude in a lysis buffer (urea 6 M in 100 mM Tris/HCl, pH = 7.5) for overnight tryptic digestion at 37 °C. As previously reported, the number of separated EVs was used as a normalization parameter for protein label-free identification and quantification [25]. Tryptic peptides were analyzed in triplicate by LC–MS/MS using the UltiMate3000 UPLC chromatographic system (Thermo Fisher Scientific, Milan, Italy) coupled to the Orbitrap Fusion Tribrid mass spectrometer (Thermo Fisher Scientific). Details of LC–MS/MS parameters are reported in our previous works [25, 26]. Briefly, the flow rate was set at 300 nL/min, with a total run time of 65 min using a chromatographic gradient from 2 to 90% of acetonitrile/water. Peptides were acquired in positive-ion polarity with data-dependent acquisition mode and MS2 sequence, using N2 as collision gas for HCD fragmentation.

Proteomics LC–MS/MS raw data were then processed using the free computational platforms MaxQuant version 1.6.10.50 and Perseus version 1.6.10.50 (Max-Planck Institute for Biochemistry, Martinsried, Germany).

and the UniProt database (released 2020_06, taxonomy Homo Sapiens, 20 588 entries), as previously described in full [27]. Proteins with a − 0.05 < p-value > 0.05 and FC > 1 were considered differentially loaded into EVs. Lastly, protein ratios (EV SAHA/EV Ctr) were used for Gene Ontology and functional enrichment analysis using the Ingenuity Pathway Analysis (IPA) tool (Qiagen, Hilden, Germany). Briefly, IPA can predict the activation (z-scores ≥ 2.0) or inhibition (z-scores ≤ − 2.0) of transcriptional regulators or downstream for the loaded dataset based on prior knowledge of expected effects from published literature citations stored in the IPA system. The proteomics data have been deposited to the ProteomeXchange Consortium via the PRIDE [28] partner repository with the dataset identifier PXD042168.

### Statistical analysis

All experiments were repeated three times (biological replicates). Statistical analysis was performed using GraphPad Prism 7 software; data were expressed as means of values and error bars represent standard deviation of the means. A two-tailed unpaired t-test was applied to assess the effects of independent variables on quantitative results. Significance was defined as a p-value < 0.05, indicated by asterisks in the figures (ns = not significant, * = p < 0.05, ** = p < 0.01, *** = p < 0.001, **** = p < 0.0001).

## Results

### SAHA modulates secretion and miRNA content in leukemia-derived EVs

**Fig. 1:**
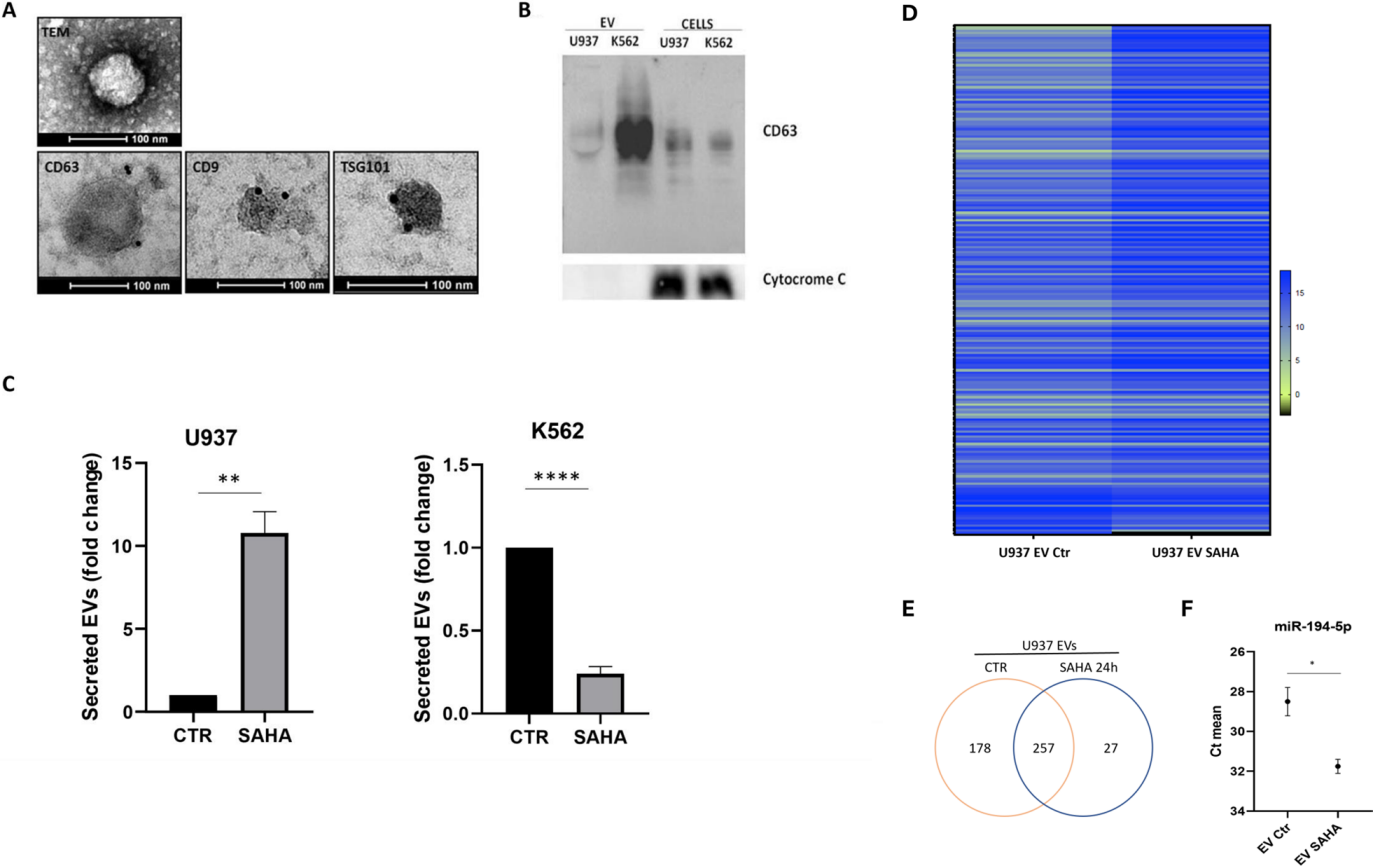
Characterization of leukemia-derived EVs and SAHA affects leukemia EV-associated miRNA profile. **A** *Top*, transmission electron microscopy micrographs of EVs isolated from U937 cells. Scale bar, 100 nm; *Bottom,* CD63, CD9, and Tsg101 analysis by immunoelectron microscopy in EVs isolated from U937 cells; **B** Western blot for CD63 and cytochrome C in U937 and K562 cells and corresponding EVs; **C** *Left*, SAHA-induced effect on EV secretion in U937 cells assessed by FACS; *Right,* SAHA induced effect on EV secretion in K562 cells assessed by FACS. Data were normalized to viable cell count and conditioned medium volume and expressed as fold change relative to the experimental control. Error bars indicate standard deviation; **D** Heat map of differentially expressed miRNAs in EVs derived from untreated vs SAHA-treated cells (Supplementary Table 1 shows the miRNA list); **E** Venn diagram of miRNAs carried by EVs derived from untreated vs SAHA-treated cells; **F** miR-194-5p sorting in U937 EVs modulated by SAHA expressed as SAHA mean. Error bars indicate standard deviation

We previously conducted a comprehensive analysis of SAHA’s effect on cell viability and apoptosis across various leukemia cell lines [11]. Briefly, we demonstrated that SAHA increased the proportion of cells in the pre-G1 phase, while enhancing Caspase-8 and −9 activity after 24 h of treatment, resulting in approximately 50% cell death in U937 cells and 40% in K562 cells (Fig. S1A). Building on these observations, we then shifted our focus to investigating the impact of SAHA on the EV secretion and composition. We isolated and characterized EVs using electron microscopy (Fig. 1A) and Western blotting (Fig. 1B). Flow cytometry analysis, optimized for stained EVs within the 100–300 nm size range, revealed that SAHA treatment increase4d EV secretion from U937 (Fig. 1C). Although SAHA exerted a cytotoxic effect on Leukemia cells [18], the enhanced EV secretion could not be attributed to an increase in apoptotic bodies, given the applied sorting threshold. Conversely, SAHA reduced EV secretion in K562 cells (Fig. 1C). Though changes in EV yield may significantly influence the tumor microenvironment, SAHA-induced intracellular molecular rearrangements [18] may be reflected in the extracellular compartment further contributing to phenotypic alterations in recipient cell, highlighting the need for molecular evaluations to investigate the malignant potential of Leukemia-derived EVs following treatment.

Accordingly, we assessed the miRNA profile of AML cells and their corresponding EVs to determine how SAHA affects miRNA loading. Treatment-induced changes in miRNA composition were observed both intracellularly and within EVs (Fig. S1 B-C). Notably, SAHA significantly altered the miRNA profile of U937 EVs (Fig. 1D, E, Table S1). Among 257 miRNAs shared between cells and vesicles, several tumor suppressors—such as miR424-5p, miR455-3p, miR-16-5p, miR-19a-3p, miR-19b-1-5p, miR-15a-5p [29–36]—were lowered in EVs following treatment (Table S1). In contrast, SAHA enhanced the loading of miRNAs associated with drug sensitivity, including miR424-5p, miR-451a, miR-153-3p, miR-17-5p, miR-21-5p, miR-27a-3p [30, 37–39]. Intriguingly, these miRNAs exhibit diverse effects on drug responsiveness, suggesting that SAHA-altered EVs may support pro-tumorigenic functions while potentially enhancing the efficacy of specific therapies. Gene Ontology (GO) analysis for the predictive targets of miRNAs differentially expressed in SAHA-modulated EV in AML, distinguishing between up- and down-regulated, reveals some peculiar biological processes and key pathways such as miRNA involvement in cancer, regulation of the G1/S transition in the mitotic cell cycle, the positive induction of apoptosis and differentiation, and the regulation of transcription by RNA pol II. These findings underscore the critical role of miRNAs in U937-derived EVs and their modulation by SAHA, prompting us to speculate on the potential impact of these changes on recipient cells. Furthermore, miR-194-5p was upregulated intracellularly upon SAHA treatment, yet its EV-associated levels were reduced (Fig. 1F; Table S1).

To explore SAHA’s impact on BCLAF1 (mRNA and protein) sorting into EVs, we analyzed specific EV subpopulations, by specifically analyzing different vesicle subpopulations (small vesicles, SVs, and large vesicles, LVs). EVs from untreated cells were designed as EV Ctr (LV Ctr and SV Ctr), while those from SAHA-treated leukemic cells were referred to as EV SAHA (LV SAHA and SV SAHA). Such deeper investigation highlighted that the reduced miR-194-5p loading was observed in both SV and LV (Supplementary Fig. 1 C-D). These findings highlight that EV content modifications induced by treatment may not directly mirror intracellular expression changes, warranting further investigation into other EV-associated molecules.

### SAHA enhances BCLAF1 secretion via EVs in leukemia cells

**Fig. 2:**
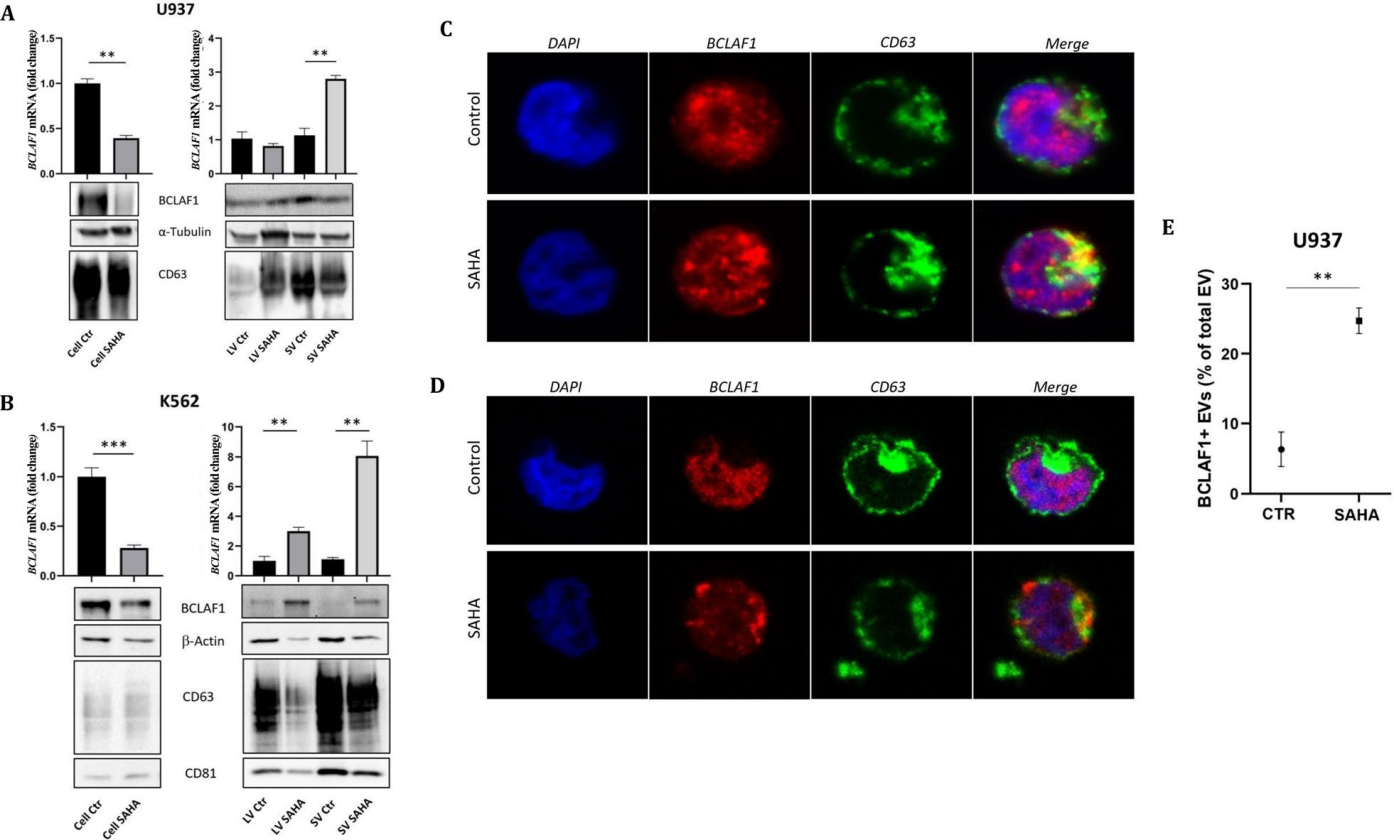
BCLAF1 quantification in leukemic cells and related EVs. **A** BCLAF1 and EV marker expression in U937 cells and cognate vesicles after 24 h SAHA treatment. *Top left*, *BCLAF1*mRNA quantification in U937 cells normalized to GAPDH expression; *Bottom left*, BCLAF1 protein quantification normalized to tubulin expression and CD63 expression in U937 cells with or without SAHA; *Top right*, *BCLAF1*mRNA quantification in U937-derived EVs normalized to Y-RNA; *Bottom right*, BCLAF1 and EV-associated marker quantification in U937-derived EVs; **B** BCLAF1 expression in K562 cells after 24 h SAHA treatment. *Top left,* mRNA quantification in K562 cells normalized to *GAPDH* mRNA expression; *Bottom left*, protein quantification normalized to β-actin and CD63 expression in K562 cells treated with or without SAHA; *Top right*, *bclaf1*mRNA quantification in K562-derived EVs normalized to Y-RNA; *Bottom right*, BCLAF1 and EV-associated marker quantification in K562-derived EVs. Error bars indicate standard deviation; **C** Immunofluorescence-based co-staining of BCLAF1 and CD63 (with DAPI for nucleus staining) in U937 cells untreated or treated with SAHA; **D** BCLAF1 and CD63 co-staining in K562 cells untreated or treated with SAHA; 40X magnification; **E** FACS-based quantification of BCLAF1-positive vesicles in EVs derived from SAHA treated vs untreated U937 cells. Data were normalized to viable cells and conditioned medium volume. Results are expressed as percentage of BCLAF1 + EVs to the total number of events. Error bars indicate standard deviation

In U937, SAHA downregulated *BCLAF1*mRNA in cells (Fig. 2A, left) yet it increased its loading into SVs. However, BCLAF1 protein levels in EVs were not significantly altered, aligning with trends observed for EV-associated markers (CD63 for small vesicles—being it a recognized endosomal marker—and α-tubulin, indicative of larger ones)(Fig. 2A, right). Particularly, LV SAHA showed increased α-tubulin levels, suggesting enhanced LV secretion (Fig. 1C, left) and resulting in greater BCLAF1 circulation.

On the other hand, in K562, SAHA induced BCLAF1 loading (both mRNA and protein) in all EV subpopulations, despite reduced EV marker detection (Fig. 2B, right), thus further proving that the treatment differently affected intra- and extra-cellular compartment content.

Furthermore, imaging approach revealed increased BCLAF1/CD63 overlap in both U937 (Fig. 2C) and K562 cells (Fig. 2D), therefore confirming the enhanced association of BCLAF1 with the extracellular compartment following SAHA treatment (observation confirmed in U937 also at cytometry level in Fig. 2E). Based on these results, we speculated that BCLAF1 might be either regarded as waste for disposal or exert functional activity within the tumor microenvironment, potentially contributing to treatment resistance.

### SAHA induces prominent changes in leukemia EV proteomics

**Fig. 3:**
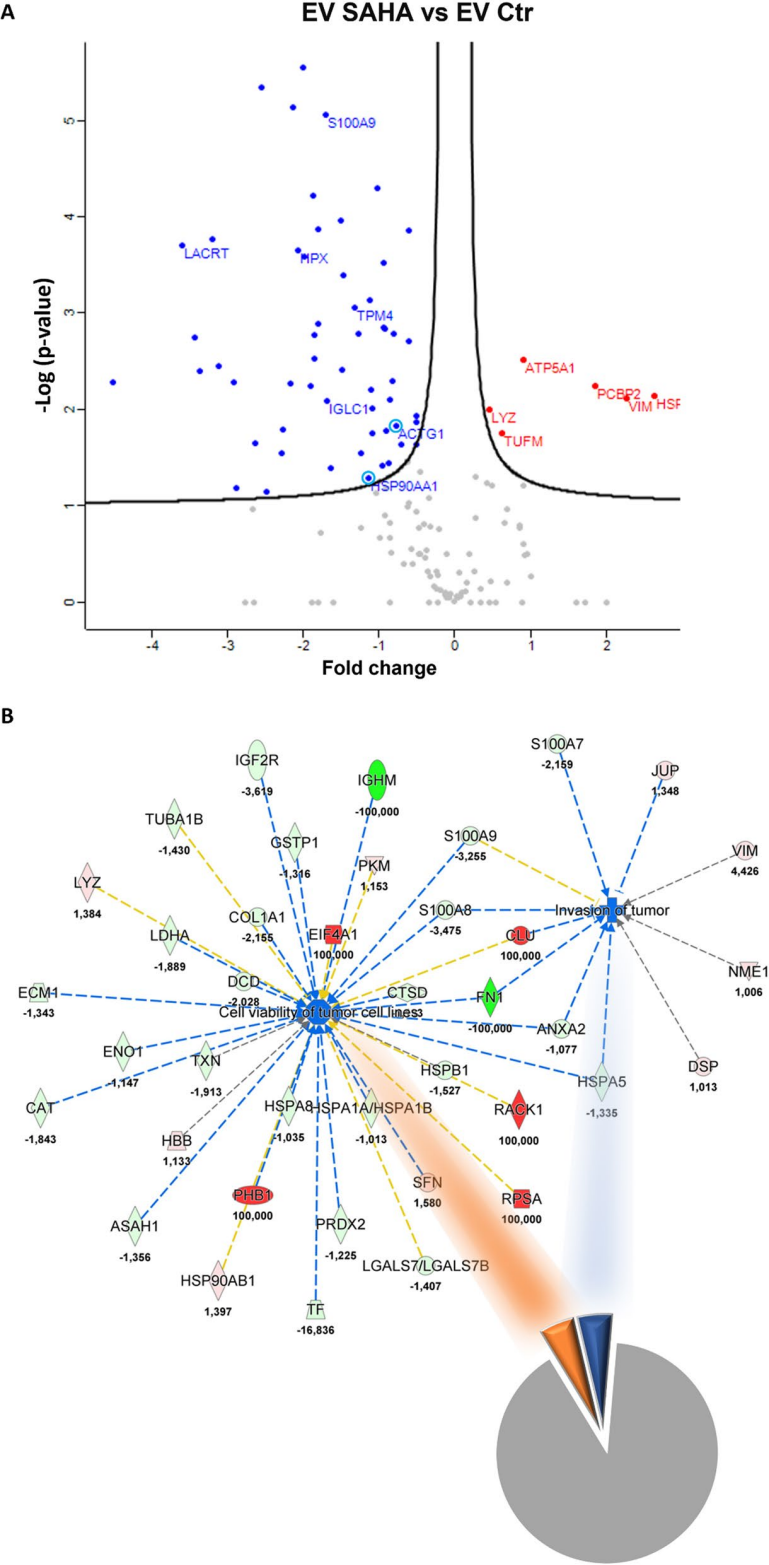
SAHA-induced changes in AML EV content and resulting biological functions predicted to be inhibited in recipient cells. **A** Volcano plot showing differentially loaded proteins in AML EVs upon SAHA treatment. Proteins most detected in EV SAHA compared to EV Ctr are in red; proteins less detected in the SAHA group compared to related Ctr are in blue. Proteins involved in drug resistance are circled (ACTG1, fold change = − 0.76; HSP90AA1, fold change = − 1.13); **B** Ingenuity Pathway Analysis showing functions predicted to be modulated in recipient cells by U937 EVs. *Bottom*, pie chart of all functions predicted to be modulated by the vesicle groups. Functions not significantly modulated in the comparison between EV SAHA vs EV Ctr are in gray. Functions predicted to be significantly inhibited in EV SAHA are in orange (“Cell viability of tumor cell lines”, 4.3%) and blue (“Invasion of tumor”, 4.3%). *Top*, proteins involved in the reported functions. The function “Invasion of tumor” showed a p-value of 6.5 × 10^–6^ and z-score of − 2.155. The function “Cell viability of tumor cell lines” showed a p-value of 1.10 × 10^–9^ and z-score of − 2.391. Activation z-score £ − 2 means the function is inhibited; z-score ≥ + 2 means the function is induced

Besides BCLAF1, we also explored the entire proteomic landscape. Proteomics analysis of U937-derived EVs detected 140 proteins in EV Ctr and 150 in EV SAHA, with 130 proteins (81.3%) shared between the two groups (Table 1). EV SAHA exhibited lower levels of actin (ACTG1) and heat shock protein 90 (HSP90) (Fig. 3), both implicated in drug resistance [40–42]. Conversely, proteins such as Cullin-3 (Cul3), associated with drug sensitivity [43], and Interleukin Enhancer Binding Factor 2 (ILF2), linked to resistance to genotoxic drugs [44], were uniquely identified in EV SAHA. Moreover, Ingenuity Pathway Analysis highlighted differential biological functions induced by the EV groups, predicting that EV SAHA could inhibit tumor invasion and viability (Table 2, Fig. 3).

**Table 1:** Proteomics in U937 EVs w/wo SAHA

T: Majority protein IDs	T: Protein names	T: Gene names	Mean CTRL	Mean SAHA	ratio	N: Razor + unique peptides	N: Unique peptides	N: Sequence coverage [%]	N: Unique + razor sequence coverage [%]	N: Unique sequence coverage [%]	N: Score	N: Intensity	N: MS/MS count	N: Deamidation (NQ) site IDs	N: Oxidation (M) site IDs	N: Deamidation (NQ) site positions	N: Oxidation (M) site positions
P0DOY3	Ig lambda-1 chain C regions	IGLC1	3024333	958543,3	0,316944	2	2	28,3	28,3	28,3	8,1553	83641000	12	0	0	0	0
O43505	Beta-1,4-glucuronyltransferase 1	B4GAT1	3597400	0	0	2	2	6,7	6,7	6,7	2,0282	2,05E+08	2	0	0	0	0
P12814	Alpha-actinin-1	ACTN1	17439,5	18951,5	1,0867	1	1	1,3	1,3	1,3	2,0368	3784700	1	0	0	0	0
O75223	Gamma-glutamylcyclotransferase	GGCT	954640	830313,3	0,869766	3	3	22,3	22,3	22,3	5,6837	57203000	12	0	0	0	0
O75629	Protein CREG1	CREG1	640396,7	356130	0,556108	1	1	9,5	9,5	9,5	4,9679	23701000	7	0	0	0	0
O95498	Vascular non-inflammatory molecule 2	VNN2	1843167	665846,7	0,361251	1	1	2,7	2,7	2,7	2,2831	1,51E+08	7	0	0	0	0
P00338	L-lactate dehydrogenase A chain	LDHA	1180014	624650	0,529358	5	4	15,4	15,4	11,7	7,0439	97451000	14	0	0	0	0
P00558	Phosphoglycerate kinase 1	PGK1	150228,7	225636,7	1,501955	4	4	16,1	16,1	16,1	7,4889	30445000	14	0	0	0	0
P00738	Haptoglobin	HP	865240	78186	0,090363	2	2	7,4	7,4	7,4	15,837	60545000	6	0	0	0	0
P01009	Alpha-1-antitrypsin	SERPINA1	371513,3	41407,33	0,111456	2	2	6,7	6,7	6,7	3,0033	27253000	6	0	0	0	0
P01023	Alpha-2-macroglobulin	A2M	65917	0	0	3	3	7,2	3,3	3,3	5,145	10129000	4	0	0	0	0
P01833	Polymeric immunoglobulin receptor	PIGR	41321,5	23055,33	0,55795	3	3	7,1	7,1	7,1	6,9351	6224100	3	0	0	0	0
P01834	Ig kappa chain C region	IGKC	20766333	7346533	0,353771	4	4	64,5	64,5	64,5	21,066	4,22E+08	20	0	0	0	0
P01857	Ig gamma-1 chain C region	IGHG1	9481267	1132213	0,119416	8	3	29,7	29,7	14,5	38,054	4,78E+08	38	0	0	0	0
P01859	Ig gamma-2 chain C region	IGHG2	871910	116103,7	0,13316	2	2	16	5,8	5,8	5,4205	38532000	5	0	0	0	0
P01860	Ig gamma-3 chain C region	IGHG3	561986,7	178555	0,317721	2	1	20,7	7,4	3,2	2,8015	34732000	5	0	0	0	0
P01871	Ig mu chain C region	IGHM	193566,7	0	0	1	1	3,5	3,5	3,5	2,1518	15448000	4	0	0	0	0
P01876	Ig alpha-1 chain C region	IGHA1	2626800	626970	0,238682	5	5	22,9	22,9	22,9	7,3161	1,56E+08	14	0	0	0	0
P02042	Hemoglobin subunit delta	HBD	0	379640	#DIV/0!	2	2	49,7	21,8	21,8	3,6179	15511000	3	0	92	0	56
P02452	Collagen alpha-1(I) chain	COL1A1	1326600	615536,7	0,463996	13	13	11,3	11,3	11,3	63,305	4,31E+08	53	0	0	0	0
P02545	Prelamin-A/C	LMNA	88461,33	70044	0,791804	4	4	8,3	8,3	8,3	8,3177	19496000	12	0	0	0	0
P02647	Apolipoprotein A-I	APOA1	909843,3	97142,5	0,106768	6	6	28,5	28,5	28,5	9,4121	52628000	12	0	0	0	0
P02656	Apolipoprotein C-III	APOC3	1154500	0	0	1	1	16,2	16,2	16,2	2,8238	20624000	5	0	0	0	0
P02671	Fibrinogen alpha chain	FGA	51598	0	0	3	3	5,8	5,8	5,8	5,494	6037000	6	0	0	0	0
P02679	Fibrinogen gamma chain	FGG	215565	0	0	2	2	7,7	7,7	7,7	2,6511	12957000	4	0	0	0	0
P02751	Fibronectin	FN1	146480	0	0	7	7	4,7	4,7	4,7	13,857	48538000	13	0	0	0	0
P02753	Retinol-binding protein 4	RBP4	2802200	288456,7	0,102939	3	3	22,4	22,4	22,4	6,115	92719000	10	0	0	0	0
P02787	Serotransferrin	TF	758360	45043,67	0,059396	8	8	17,2	15,5	15,5	11,988	98819000	16	0	0	0	0
P02788	Lactotransferrin	LTF	1235633	337136,7	0,272845	10	10	21,5	18,6	18,6	19,323	2,22E+08	27	0	0	0	0
P02790	Hemopexin	HPX	681426,7	162616,7	0,238641	2	2	8	8	8	5,1183	58239000	9	0	0	0	0
P04040	Catalase	CAT	849520	460890	0,54253	8	8	20,7	20,7	20,7	16,413	1,14E+08	20	0	0	0	0
P04075	Fructose-bisphosphate aldolase A	ALDOA	258683,3	247090	0,955183	4	4	19,5	19,5	19,5	6,8045	34898000	9	0	0	0	0
P04083	Annexin A1	ANXA1	232215	162730	0,700773	2	2	9,5	9,5	9,5	3,8673	19052000	7	0	0	0	0
P04259	Keratin, type II cytoskeletal 6B	KRT6B	199320	339326,7	1,702422	2	2	54,4	5,5	5,5	4,8718	42499000	6	0	0	0	0
P04406	Glyceraldehyde-3-phosphate dehydrogenase	GAPDH	7801833	4441367	0,569272	12	12	47,2	47,2	47,2	55,304	6,24E+08	46	0	98	0	105
P04439	HLA class I histocompatibility antigen, A-3 alpha chain	HLA-A	0	214133,3	#DIV/0!	2	2	10,4	10,4	10,4	8,5546	16661000	6	0	0	0	0
P04792	Heat shock protein beta-1	HSPB1	730773,3	478560	0,654868	3	3	28,3	28,3	28,3	6,3727	50792000	10	0	0	0	0
P05089	Arginase-1	ARG1	1303387	939136,7	0,720536	7	7	32	32	32	10,194	1,14E+08	15	0	99	0	212
P05090	Apolipoprotein D	APOD	7313667	3748600	0,512547	7	7	36	36	36	16,965	2,99E+08	29	103	0	58	0
P05109	Protein S100-A8	S100A8	29468333	8481233	0,287808	5	5	33,3	33,3	33,3	158,96	9,11E+08	30	0	0	0	0
P05141	ADP/ATP translocase 2	SLC25A5	352896,7	145526,3	0,412377	2	2	7,4	7,4	7,4	3,255	28410000	7	0	0	0	0
P06576	ATP synthase subunit beta, mitochondrial	ATP5B	678429,7	942983	1,38995	6	6	15,7	15,7	15,7	20,732	1,41E+08	17	0	0	0	0
P06702	Protein S100-A9	S100A9	40364333	12401667	0,307243	4	4	35,1	35,1	35,1	17,919	1,27E+09	27	0	0	0	0
P06733	Alpha-enolase	ENO1	2829867	2466133	0,871466	13	13	40,8	40,8	40,8	102,78	3,5E+08	50	0	0	0	0
P67936	Tropomyosin alpha-4 chain	TPM4	159100	64219,67	0,403643	1	1	6	6	6	1,875	10049000	3	0	0	0	0
P07195	L-lactate dehydrogenase B chain	LDHB	775513,7	636080	0,820205	4	4	18,6	15	15	6,455	71991000	14	0	0	0	0
P07339	Cathepsin D	CTSD	1184080	992180	0,837933	7	7	23,8	23,8	23,8	11,785	1,24E+08	15	0	0	0	0
P07355	Annexin A2	ANXA2	4015100	3728667	0,928661	17	17	56	56	56	149,79	5,34E+08	69	0	0	0	0
P07437	Tubulin beta chain	TUBB	2327600	2506500	1,07686	14	4	36,7	36,7	13,5	32,311	2,9E+08	38	0	0	0	0
P07900	Heat shock protein HSP 90-alpha	HSP90AA1	74588	33384	0,447579	1	1	4	2	2	2,2077	7126100	1	0	0	0	0
P08123	Collagen alpha-2(I) chain	COL1A2	293849	31305,67	0,106537	4	4	3,7	3,7	3,7	6,0019	67307000	9	0	0	0	0
P08195	4F2 cell-surface antigen heavy chain	SLC3A2	960820	984966,7	1,025131	9	9	23	23	23	31,238	1,69E+08	21	0	0	0	0
P08238	Heat shock protein HSP 90-beta	HSP90AB1	191737	267940	1,397435	4	3	7,7	7,7	5,8	7,6134	45508000	11	0	0	0	0
P08603	Complement factor H	CFH	51551	0	0	1	1	1,5	1,5	1,5	1,7397	7143900	2	0	0	0	0
P08670	Vimentin	VIM	127883,3	565976,7	4,425727	7	7	21,5	20	20	25,887	70774000	21	0	109	0	183
P08865	40S ribosomal protein SA	RPSA	0	570986,7	#DIV/0!	3	3	13,9	13,9	13,9	3,311	24469000	4	0	0	0	0
P09211	Glutathione S-transferase P	GSTP1	970100	737076,7	0,759795	4	4	31	31	31	7,2895	56337000	11	0	0	0	0
P0C0L5	Complement C4-B	C4B	234570	36175,67	0,154221	1	1	3,2	1,3	1,3	2,4187	65792000	2	0	0	0	0
P62987	Ubiquitin-60S ribosomal protein L40	UBA52	1361470	1496540	1,099209	3	3	26,6	26,6	26,6	12,052	51444000	6	0	0	0	0
P0DMV9	Heat shock 70 kDa protein 1B	HSPA1B	53216,33	52536	0,987216	2	2	6,7	4,7	4,7	3,1171	10152000	3	0	0	0	0
P0DP25			2122050	382903,3	0,18044	2	2	22,1	22,1	22,1	3,2065	48535000	6	0	0	0	0
P10599	Thioredoxin	TXN	3276633	1712567	0,52266	3	3	19	19	19	70,383	1,05E+08	9	0	0	0	0
P10809	60 kDa heat shock protein, mitochondrial	HSPD1	133788,3	760810	5,686669	12	12	31,4	31,4	31,4	21,873	85882000	19	0	110	0	145
P10909	Clusterin	CLU	0	75574,33	#DIV/0!	3	3	9,1	9,1	9,1	3,8193	5795700	5	0	0	0	0
P11021	78 kDa glucose-regulated protein	HSPA5	230770	172870	0,749101	4	4	9,3	9,3	9,3	5,8456	29404000	8	0	0	0	0
P11142	Heat shock cognate 71 kDa protein	HSPA8	673410	650916,7	0,966598	7	6	15	15	13	12,633	1,31E+08	25	0	0	0	0
P11717	Cation-independent mannose-6-phosphate receptor	IGF2R	14463,33	3996,467	0,276317	2	2	1,1	1,1	1,1	1,799	7033300	1	0	0	0	0
P12109	Collagen alpha-1(VI) chain	COL6A1	398336,7	91040	0,22855	3	3	4,3	4,3	4,3	6,5088	79278000	15	0	0	0	0
P12273	Prolactin-inducible protein	PIP	39583000	25791000	0,651568	9	9	63	63	63	24,092	1,77E+09	84	0	0	0	0
P12724	Eosinophil cationic protein	RNASE3	1876367	771363,3	0,411094	3	3	20	20	20	24,174	63545000	11	0	0	0	0
P14618	Pyruvate kinase PKM	PKM	656820	757583,3	1,153411	10	10	28,1	28,1	28,1	18,813	1,4E+08	25	0	0	0	0
P14923	Junction plakoglobin	JUP	1933900	2607333	1,348226	20	20	36,4	36,4	36,4	45,312	6,27E+08	69	0	111	0	193
P15531	Nucleoside diphosphate kinase A	NME1	286900	288746,7	1,006437	2	2	19,1	19,1	19,1	2,4048	12960000	2	0	0	0	0
P15924	Desmoplakin	DSP	816183,3	826963,3	1,013208	49	49	21,9	21,9	21,9	186,47	8,04E+08	130	0	0	0	0
P18206	Vinculin	VCL	93403	10588,73	0,113366	2	2	2,5	2,5	2,5	3,5375	23398000	4	0	0	0	0
P35321	Cornifin-A	SPRR1A	897543,3	609890	0,67951	2	2	33,7	33,7	33,7	2,8872	31656000	7	0	0	0	0
P22531	Small proline-rich protein 2E	SPRR2E	6261967	3895300	0,622057	5	5	79,2	79,2	79,2	39,583	1,83E+08	29	0	0	0	0
P22626	Heterogeneous nuclear ribonucleoproteins A2/B1	HNRNPA2B1	0	255576,7	#DIV/0!	2	2	9,9	9,9	9,9	3,0291	16596000	3	0	0	0	0
P22735	Protein-glutamine gamma-glutamyltransferase K	TGM1	324880	175636,7	0,54062	4	4	7,6	7,6	7,6	9,1441	70573000	11	0	0	0	0
P23284	Peptidyl-prolyl cis–trans isomerase B	PPIB	901680	533236,7	0,591381	2	2	12,5	12,5	12,5	3,9061	51657000	9	0	0	0	0
P23528	Cofilin-1	CFL1	1400967	1103867	0,787932	3	3	31,9	31,9	31,9	7,7265	75145000	15	0	0	0	0
P25311	Zinc-alpha-2-glycoprotein	AZGP1	6221800	2839633	0,456401	10	10	39,6	39,6	39,6	89,875	4,89E+08	57	0	0	0	0
P25705	ATP synthase subunit alpha, mitochondrial	ATP5A1	222403,3	422236,7	1,898518	5	5	13	13	13	8,4559	59951000	15	0	0	0	0
P26038	Moesin	MSN	243870	134134	0,550023	6	6	13	13	13	7,209	37422000	12	0	0	0	0
P27482	Calmodulin-like protein 3	CALML3	548010	118752,7	0,216698	1	1	11,4	11,4	11,4	2,2242	15975000	3	0	0	0	0
P29401	Transketolase	TKT	53716	112712	2,098295	2	2	5,5	5,5	5,5	2,7178	8321500	4	0	0	0	0
P29508	Serpin B3	SERPINB3	3112867	1646333	0,52888	12	5	39,2	39,2	21,5	45,387	3,14E+08	57	0	0	0	0
P31025	Lipocalin-1	LCN1	11264000	1926100	0,170996	5	5	43,8	43,8	43,8	13,852	3,17E+08	20	0	0	0	0
P31151	Protein S100-A7	S100A7	9065467	4199533	0,463245	4	4	35,6	35,6	35,6	6,5448	2,79E+08	13	0	0	0	0
P31943	Heterogeneous nuclear ribonucleoprotein H	HNRNPH1	0	161783,3	#DIV/0!	3	3	10,9	10,9	10,9	5,1523	8736300	3	0	0	0	0
P31944	Caspase-14	CASP14	2847867	756770	0,265732	5	5	26,9	26,9	26,9	64,76	1,62E+08	18	0	0	0	0
P31947	14-3-3 protein sigma	SFN	186640	294930	1,580208	3	3	15,7	15,7	15,7	6,4496	18396000	8	0	0	0	0
P32119	Peroxiredoxin-2	PRDX2	995530,3	812433,3	0,816081	3	3	25,3	19,7	19,7	3,8657	70510000	10	0	0	0	0
P35232	Prohibitin	PHB	0	106717	#DIV/0!	2	2	8,8	8,8	8,8	2,1655	6662000	4	0	0	0	0
P47929	Galectin-7	LGALS7	2184867	1552700	0,710661	4	4	36	36	36	10,153	1,12E+08	14	0	0	0	0
P48594	Serpin B4	SERPINB4	284826,7	163788,7	0,575047	2	2	25,4	7,7	7,7	2,4818	28263000	5	0	0	0	0
P49411	Elongation factor Tu, mitochondrial	TUFM	108802	165426,7	1,520438	3	3	10,8	10,8	10,8	5,2651	24681000	5	0	0	0	0
P50990	T-complex protein 1 subunit theta	CCT8	0	22514,5	#DIV/0!	1	1	2,7	2,7	2,7	1,7523	1666100	2	0	0	0	0
P51571	Translocon-associated protein subunit delta	SSR4	0	522070	#DIV/0!	2	2	17,3	17,3	17,3	4,3778	12108000	4	0	0	0	0
P56470	Galectin-4	LGALS4	361494,7	384540	1,06375	2	2	9,3	9,3	9,3	4,8501	31333000	7	0	0	0	0
P58107	Epiplakin	EPPK1	0	4834,633	#DIV/0!	1	1	2,2	2,2	2,2	2,3164	3959600	3	0	0	0	0
P60174	Triosephosphate isomerase	TPI1	452483,3	190602,7	0,421237	3	3	17,3	17,3	17,3	4,7027	32797000	6	0	0	0	0
P60660	Myosin light polypeptide 6	MYL6	388650	582590	1,499009	2	2	18,5	18,5	18,5	3,7237	25251000	4	0	0	0	0
P63261	Actin, cytoplasmic 2	ACTG1	18784333	10945333	0,582684	21	10	65,9	65,9	40,5	130,06	1,78E+09	75	0	0	0	0
P60842	Eukaryotic initiation factor 4A-I	EIF4A1	0	281923,3	#DIV/0!	2	2	6,4	6,4	6,4	2,9604	24389000	3	0	0	0	0
P60953	Cell division control protein 42 homolog	CDC42	0	445463,3	#DIV/0!	1	1	11	11	11	3,4855	15649000	3	0	0	0	0
P61626	Lysozyme C	LYZ	20341000	28152000	1,384003	8	8	55,4	55,4	55,4	63,377	1,31E+09	37	0	0	0	0
P61978	Heterogeneous nuclear ribonucleoprotein K	HNRNPK	0	186296,7	#DIV/0!	3	3	10,6	10,6	10,6	3,8117	12854000	5	0	0	0	0
P62263	40S ribosomal protein S14	RPS14	1518602	1546767	1,018547	5	5	19,9	19,9	19,9	7,4129	36784000	7	0	0	0	0
P62318	Small nuclear ribonucleoprotein Sm D3	SNRPD3	406245	289036,7	0,711484	1	1	7,9	7,9	7,9	2,1014	10078000	3	0	0	0	0
P63267	Actin, gamma-enteric smooth muscle	ACTG2	0	330633,3	#DIV/0!	4	4	47,1	21,8	21,8	9,6904	19838000	5	0	0	0	0
P62805	Histone H4	HIST1H4A	2703533	2784167	1,029825	5	5	51,5	51,5	51,5	8,4809	98778000	13	0	0	0	0
P62888	60S ribosomal protein L30	RPL30	355340	198566,7	0,558808	2	2	24,3	24,3	24,3	2,5793	9970300	2	0	0	0	0
P62913	60S ribosomal protein L11	RPL11	216583,3	171705	0,79279	1	1	7,9	7,9	7,9	1,78	9931600	1	0	0	0	0
P62937	Peptidyl-prolyl cis–trans isomerase A	PPIA	1560307	2416733	1,548884	4	4	31,5	31,5	31,5	6,6593	1,19E+08	13	0	0	0	0
P63104	14-3-3 protein zeta/delta	YWHAZ	741813,3	456110	0,614858	4	4	18,4	18,4	18,4	44,405	50313000	11	0	0	0	0
P63244	Guanine nucleotide-binding protein subunit beta-2-like 1	GNB2L1	0	250430	#DIV/0!	3	3	12	12	12	4,4906	18331000	6	0	0	0	0
P68104	Elongation factor 1-alpha 1	EEF1A1	2753900	1548533	0,562306	7	7	24,2	24,2	24,2	45,132	2,71E+08	30	0	0	0	0
P68363	Tubulin alpha-1B chain	TUBA1B	4697133	3284300	0,699214	10	3	35,9	35,9	9,8	30,944	5,03E+08	45	0	0	0	0
P68371	Tubulin beta-4B chain	TUBB4B	230970	280496,7	1,214429	1	1	25,8	2,7	2,7	5,953	26069000	6	0	0	0	0
Q71DI3	Histone H3.2	HIST2H3A	3450500	2862367	0,829551	2	2	13,2	13,2	13,2	2,4243	75754000	10	0	0	0	0
P68871	Hemoglobin subunit beta	HBB	7204600	8161667	1,132841	7	3	55,8	49	27,9	43,377	5,07E+08	26	0	153	0	56
P69905	Hemoglobin subunit alpha	HBA1	28117667	13499667	0,480113	8	5	70,4	70,4	47,9	58,093	9,99E+08	32	0	0	0	0
P81605	Dermcidin	DCD	18175000	8963300	0,493166	4	4	33,6	33,6	33,6	43,114	4,07E+08	12	0	0	0	0
Q01469	Fatty acid-binding protein, epidermal	FABP5	5596933	3409333	0,609143	8	8	73,3	73,3	73,3	26,24	2,16E+08	28	0	0	0	0
Q02241	Kinesin-like protein KIF23	KIF23	118891,7	0	0	3	3	3,9	3,9	3,9	6,3688	17834000	6	0	0	0	0
Q02413	Desmoglein-1	DSG1	2413633	1697467	0,703283	21	21	34,7	34,7	34,7	59,708	5,06E+08	55	0	0	0	0
Q06828	Fibromodulin	FMOD	198120	57155,67	0,28849	1	1	3,5	3,5	3,5	3,4136	9955800	4	0	0	0	0
Q06830	Peroxiredoxin-1	PRDX1	2756467	2883300	1,046013	9	8	38,7	38,7	33,2	13,242	2,54E+08	28	0	0	0	0
Q08188	Protein-glutamine gamma-glutamyltransferase E	TGM3	1219600	763213,3	0,62579	7	7	18,8	18,8	18,8	20,056	2,38E+08	28	0	0	0	0
Q08554	Desmocollin-1	DSC1	1331333	873716,7	0,656272	11	11	17,6	17,6	17,6	49,493	3,18E+08	42	0	167	0	453
Q12905	Interleukin enhancer-binding factor 2	ILF2	0	91951,67	#DIV/0!	2	2	7,9	7,9	7,9	2,9966	5241300	2	0	0	0	0
Q13510	Acid ceramidase	ASAH1	128174,7	94507,33	0,737332	1	1	5,8	5,8	5,8	2,962	16033000	4	0	0	0	0
Q13618	Cullin-3	CUL3	0	115725	#DIV/0!	1	1	1,8	1,8	1,8	1,8572	10184000	2	0	0	0	0
Q13835	Plakophilin-1	PKP1	209325	193980	0,926693	5	5	9,1	9,1	9,1	61,441	46027000	16	0	0	0	0
Q13867	Bleomycin hydrolase	BLMH	179283,3	182646,7	1,01876	2	2	7,3	7,3	7,3	2,4391	28231000	5	0	0	0	0
Q14574	Desmocollin-3	DSC3	140909,7	132313,3	0,938994	2	2	3,2	3,2	3,2	4,557	40164000	10	0	0	0	0
Q15365	Poly(rC)-binding protein 1	PCBP1	328040	615710	1,876936	5	3	20,8	20,8	12,1	22,361	48131000	12	0	171	0	137
Q15366	Poly(rC)-binding protein 2	PCBP2	124795	435770	3,491887	2	2	15,9	7,4	7,4	2,329	28589000	2	0	171	0	137
Q15517	Corneodesmosin	CDSN	0	116080	#DIV/0!	2	2	7,9	7,9	7,9	2,1572	3482400	3	0	0	0	0
Q16610	Extracellular matrix protein 1	ECM1	208105	155010	0,744864	2	2	5,2	5,2	5,2	2,626	27318000	4	0	0	0	0
Q3V6T2	Girdin	CCDC88A	56381,33	20425,67	0,362277	2	2	1,2	1,2	1,2	2,0471	21660000	1	0	0	0	0
Q3ZCM7	Tubulin beta-8 chain	TUBB8	498250	361430	0,725399	1	1	15,1	7,2	7,2	2,8385	44352000	4	0	102	0	267
Q494V2	Coiled-coil domain-containing protein 37	CCDC37	96047,5	0	0	1	1	1,1	1,1	1,1	1,8333	4994400	0	0	0	0	0
Q53RT3	Retroviral-like aspartic protease 1	ASPRV1	762270	396190	0,51975	1	1	3,8	3,8	3,8	1,8044	38229000	6	0	0	0	0
Q5T749	Keratinocyte proline-rich protein	KPRP	4443733	5344000	1,202592	18	18	43,4	43,4	43,4	49,389	7,34E+08	79	0	0	0	0
Q5T750	Skin-specific protein 32	XP32	5092167	3278067	0,643747	5	5	20,4	20,4	20,4	7,8885	2,76E+08	23	0	0	0	0
Q5T7P3	Late cornified envelope protein 1B	LCE1B	5337300	2212167	0,414473	1	1	38,1	38,1	38,1	4,9208	34622000	5	0	0	0	0
Q6UWP8	Suprabasin	SBSN	103502	73861	0,713619	3	3	16,6	16,6	16,6	8,2188	13715000	4	0	0	0	0
Q6ZVX7	F-box only protein 50	NCCRP1	329156,7	358696,7	1,089744	2	2	8	8	8	4,057	24763000	6	0	0	0	0
Q8WVV4	Protein POF1B	POF1B	263826,7	281130	1,065586	6	6	13,9	13,9	13,9	21,679	44142000	11	0	0	0	0
Q92841	Probable ATP-dependent RNA helicase DDX17	DDX17	0	43555	#DIV/0!	2	2	3,3	3,3	3,3	1,8434	3223100	1	0	0	0	0
Q96DR8	Mucin-like protein 1	MUCL1	68797667	17198000	0,249979	2	2	13,3	13,3	13,3	3,397	5,16E+08	9	143	0	85	0
Q96P63	Serpin B12	SERPINB12	371250	699460	1,884067	9	9	27,7	27,7	27,7	16,385	64243000	13	0	0	0	0
Q96QA5	Gasdermin-A	GSDMA	313646,7	230080	0,733564	3	3	8,3	8,3	8,3	8,9861	39148000	11	0	0	0	0
Q9BV38	WD repeat-containing protein 18	WDR18	0	29124,5	#DIV/0!	2	2	4,4	4,4	4,4	2,7211	1106700	2	0	0	0	0
Q9GZZ8	Extracellular glycoprotein lacritin	LACRT	6958600	572235	0,082234	2	2	16,7	16,7	16,7	7,6234	88081000	4	0	0	0	0
Q9NZT1	Calmodulin-like protein 5	CALML5	2405400	1177287	0,489435	4	4	46,6	46,6	46,6	12,441	96733000	10	0	0	0	0
Q9UGM3	Deleted in malig0t brain tumors 1 protein	DMBT1	50616	13941	0,275427	1	1	5,2	5,2	5,2	3,0999	15097000	1	0	0	0	0
S4R460		IGHV3OR16-9	1274167	323823,3	0,254145	1	1	19,8	19,8	19,8	9,4025	23970000	6	0	0	0	0

Venn Diagram in U937 EVs		
Unique in EV SAHA	P02042	HBD
	P04439	HLA-A
	P08865	RPSA
	P10909	CLU
	P22626	HNRNPA2B1
	P31943	HNRNPH1
	P35232	PHB
	P50990	CCT8
	P51571	SSR4
	P58107	EPPK1
	P60842	EIF4A1
	P60953	CDC42
	P61978	HNRNPK
	P63267	ACTG2
	P63244	GNB2L1
	Q12905	ILF2
	Q13618	CUL3
	Q15517	CDSN
	Q92841	DDX17
	Q9BV38	WDR18
Unique in EV CTR	O43505	B3GNT1
	P01023	A2M
	P01871	
	P02656	APOC3
	P02671	FGA
	P02679	FGG
	P02751	FN1
	P08603	CFH
	Q02241	KIF23
	Q494V2	CCDC37
Shared between EV CTR and SAHA	P02787	TF
	Q9GZZ8	LACRT
	P00738	HP
	P02753	RBP4
	P08123	COL1A2
	P02647	APOA1
	P01009	SERPINA1
	P18206	VCL
	P01857	
	P01859	
	P0C0L5	C4B
	P31025	LCN1
	P0DP25	CALM3
	P27482	CALML3
	P12109	COL6A1
	P02790	HPX
	P01876	
	Q96DR8	MUCL1
	S4R460	ENSP00000474135
	P31944	CASP14
	P02788	LTF
	Q9UGM3	DMBT1
	P11717	IGF2R
	P05109	S100A8
	Q06828	FMOD
	P06702	S100A9
	P0DOY3	
	P01860	
	P01834	
	O95498	VNN2
	Q3V6T2	CCDC88A
	P67936	TPM4
	P12724	RNASE3
	P05141	SLC25A5
	Q5T7P3	LCE1B
	P60174	TPI1
	P07900	HSP90AA1
	P25311	AZGP1
	P31151	S100A7
	P02452	COL1A1
	P69905	HBA2
	Q9NZT1	CALML5
	P81605	DCD
	P05090	APOD
	Q53RT3	ASPRV1
	P10599	TXN
	P29508	SERPINB3
	P00338	LDHA
	P22735	TGM1
	P04040	CAT
	P26038	MSN
	O75629	CREG1
	P01833	PIGR
	P62888	RPL30
	P68104	EEF1A1
	P04406	GAPDH
	P48594	SERPINB4
	P63261	ACTG1
	P23284	PPIB
	Q01469	FABP5
	P63104	YWHAZ
	P22531	SPRR2E
	Q08188	TGM3
	Q5T750	C1orf68
	P12273	PIP
	P04792	HSPB1
	Q08554	DSC1
	P35321	SPRR1A
	P68363	TUBA1B
	P04083	ANXA1
	Q02413	DSG1
	P47929	LGALS7B
	P62318	SNRPD3
	Q6UWP8	SBSN
	P05089	ARG1
	Q3ZCM7	TUBB8
	Q96QA5	GSDMA
	Q13510	ASAH1
	Q16610	ECM1
	P11021	HSPA5
	P09211	GSTP1
	P23528	CFL1
	P02545	LMNA
	P62913	RPL11
	P32119	PRDX2
	P07195	LDHB
	Q71DI3	HIST2H3D
	P07339	CTSD
	O75223	GGCT
	P06733	ENO1
	Q13835	PKP1
	P07355	ANXA2
	Q14574	DSC3
	P04075	ALDOA
	P11142	HSPA8
	P0DMV9	HSPA1B
	P15531	NME1
	P15924	DSP
	P62263	RPS14
	Q13867	BLMH
	P08195	SLC3A2
	P62805	HIST1H4F
	Q06830	PRDX1
	P56470	LGALS4
	Q8WVV4	POF1B
	P07437	TUBB
	P12814	ACTN1
	Q6ZVX7	NCCRP1
	P62987	UBA52
	P68871	HBB
	P14618	PKM
	Q5T749	KPRP
	P68371	TUBB4B
	P14923	JUP
	P61626	LYZ
	P06576	ATP5B
	P08238	HSP90AB1
	P60660	MYL6
	P00558	PGK1
	P49411	TUFM
	P62937	PPIA
	P31947	SFN
	P04259	KRT6B
	Q15365	PCBP1
	Q96P63	SERPINB12
	P25705	ATP5A1
	P29401	TKT
	Q15366	PCBP2
	P08670	VIM
	P10809	HSPD1

**Table 2:** Downstream analysis by IPA tool—U937 EVs

Function	p-value	Activation state	Activationz-score	Proteins involved (n)
Invasion of tumor	6.50E-06	Decreased	− 2.155	11
Cell viability of tumor cell lines	1.10E-09	Decreased	− 2.391	34
Immune response of cells	6.13E-08	–	− 1.895	23
Activation of leukocytes	1.07E-06	–	− 1.226	23
Complement activation	4.37E-11	–	− 1.131	12
Cell movement of mononuclear leukocytes	7.34E-07	–	− 0.772	19
Proliferation of mononuclear leukocytes	5.34E-10	–	− 0.623	29
Activation of myeloid cells	6.34E-06	–	− 0.623	14
Activation of phagocytes	3.82E-06	–	− 0.587	15
Proliferation of lymphatic system cells	9.74E-09	–	− 0.508	28
Proliferation of lymphocytes	3.18E-08	–	− 0.508	26
Migration of mononuclear leukocytes	1.23E-05	–	− 0.108	15
Proliferation of immune cells	9.71E-11	–	− 0.073	31
Adhesion of myeloid cells	1.69E-07	–	− 0.024	11
Migration of granulocytes	1.27E-05	–	0	9
Adhesion of granulocytes	4.38E-08	–	0.017	10
Migration of neutrophils	2.06E-05	–	0.049	8
Cell movement of lymphocytes	6.06E-06	–	0.051	16
Cell proliferation of T lymphocytes	1.72E-07	–	0.089	22
Migration of phagocytes	5.02E-06	–	− 0.472	13
Angiogenesis	3.72E-10	–	− 1.784	35
Cell movement of tumor cell lines	2.37E-12	–	− 1.677	41
Migration of tumor cell lines	3.75E-13	–	− 1.414	39
Invasion of cells	4.46E-13	–	− 1.352	42
Invasion of tumor cell lines	8.50E-12	–	− 1.156	36
Growth of tumor	1.03E-05	–	− 1.107	27
Colony formation of cells	1.98E-07	–	− 0.753	21
Colony formation of tumor cell lines	1.06E-07	–	− 0.688	17
Cell proliferation of tumor cell lines	3.03E-11	–	− 0.257	53
Invasive tumor	1.76E-11	–	0.095	42
Metastasis	4.01E-10	–	0.095	36
Advanced malignant tumor	1.17E-09	–	0.095	37
Development of tumor cell lines	7.74E-08	–	− 0.494	19
Cell movement of endothelial cells	5.06E-06	–	− 0.442	15
Proliferation of vascular cells	6.30E-06	–	− 0.061	13
Migration of endothelial cells	7.75E-06	–	− 0.56	14
Apoptosis of leukemia cell lines	3.06E-06	–	− 0.389	13
Apoptosis of tumor cell lines	1.42E-14	–	− 1.054	48
Cell survival	6.85E-17	–	− 1.333	57
Organization of actin filaments	2.33E-07	–	− 0.747	9
Aggregation of blood platelets	1.48E-06	–	− 0.555	11

Most relevant biological functions predicted to be modulated by U937 EVs (SAHA vs CTR) in receiving cells. z-score £ − 2 indicates decreased function activation induced by EV SAHA compared to EV CTR. z-score ^3^ 2 indicates increased function activation induced by EV SAHA compared to EV CTR

Regarding K562, EV SAHA contained 234 proteins compared to 124 in EV Ctr, with 100 proteins (38.8%) shared (Table 3). Bioinformatic predictions suggested that EV SAHA might enhance pro-tumoral functions (Table 4). However, proteins like 60S ribosomal protein L11 (RPL11), which sensitize cells to therapeutic agents [36], were more abundant in EV SAHA.

**Table 3:** Proteomics in K562 EVs w/wo SAHA

T: Majority protein IDs	T: Protein names	T: Gene names	Mean CTRL	Mean SAHA	ratio	N: Razor + unique peptides	N: Unique peptides	N: Sequence coverage [%]	N: Unique + razor sequence coverage [%]	N: Unique sequence coverage [%]	N: Score	N: Intensity	N: MS/MS count	N: Deamidation (NQ) site IDs	N: Oxidation (M) site IDs	N: Deamidation (NQ) site positions
P62258	14-3-3 protein epsilon	YWHAE	94237	199653,3333	2,118629979	4	4	27,1	23,1	23,1	9,1403	13386000	7	0	0	0
P61981	14-3-3 protein gamma	YWHAG	0	129858	#DIV/0!	2	1	16,2	12,1	5,7	3,3896	6233200	3	0	0	0
P31947	14-3-3 protein sigma	SFN	358796,6667	2326666,667	6,484638467	10	9	47,2	47,2	43,1	50,455	1,29E+08	34	0	0	0
P63104	14-3-3 protein zeta/delta	YWHAZ	384766,6667	1076846,667	2,798700511	6	6	30,6	26,5	26,5	74,935	61387000	25	0	0	0
P62277	40S ribosomal protein S13	RPS13	350410	0	0	2	2	16,6	16,6	16,6	2,6349	9461100	5	0	0	0
P62263	40S ribosomal protein S14	RPS14	336390	0	0	3	3	23,2	23,2	23,2	6,5422	3357700	3	0	135	0
P62249	40S ribosomal protein S16	RPS16	164446,6667	72070	0,438257591	2	2	14,4	14,4	14,4	4,1142	7012400	8	0	0	0
P62269	40S ribosomal protein S18	RPS18	271048	0	0	2	2	15,1	15,1	15,1	4,0816	9426600	6	0	0	0
P62854	40S ribosomal protein S26	RPS26	824930	614585	0,745014729	2	2	20,9	20,9	20,9	4,8418	18520000	8	0	0	0
P62857	40S ribosomal protein S28	RPS28	400050	358610	0,896412948	1	1	17,4	17,4	17,4	7,4765	6827900	7	0	0	0
P23396	40S ribosomal protein S3	RPS3	51770,33333	164602,3333	3,179472156	3	3	16,5	16,5	16,5	5,0499	12333000	10	0	0	0
P61247	40S ribosomal protein S3a	RPS3A	0	53970,5	#DIV/0!	3	3	14,8	14,8	14,8	4,3054	3152700	4	0	0	0
P62701	40S ribosomal protein S4, X isoform	RPS4X	129309,6667	0	0	3	3	12,5	12,5	12,5	6,2391	6206900	5	0	0	0
P62081	40S ribosomal protein S7	RPS7	0	78364,5	#DIV/0!	2	2	11,9	11,9	11,9	3,2389	1567300	3	0	0	0
P08865	40S ribosomal protein SA	RPSA	58135	502306,6667	8,640348614	3	3	19,3	19,3	19,3	7,5656	21857000	11	0	0	0
P49189	4-trimethylaminobutyraldehyde dehydrogenase	ALDH9A1	0	146470	#DIV/0!	4	4	11,7	11,7	11,7	9,3656	10985000	7	0	0	0
P10809	60 kDa heat shock protein, mitochondrial	HSPD1	0	72371,5	#DIV/0!	4	4	12,6	12,6	12,6	11,71	5849400	5	0	0	0
P27635	60S ribosomal protein L10	RPL10	0	95124,66667	#DIV/0!	3	3	24,3	24,3	24,3	5,3719	3010900	7	0	0	0
P62913	60S ribosomal protein L11	RPL11	96445,96667	113760	1,179520554	1	1	7,9	7,9	7,9	2,4192	6306100	6	0	0	0
P30050	60S ribosomal protein L12	RPL12	0	96624,5	#DIV/0!	1	1	9,1	9,1	9,1	2,4522	1739200	2	0	0	0
P26373	60S ribosomal protein L13	RPL13	205680	95406	0,463856476	1	1	5,2	5,2	5,2	7,8965	5419500	5	0	0	0
P18621	60S ribosomal protein L17	RPL17	289766,6667	0	0	2	2	16,3	16,3	16,3	2,8429	7823700	3	0	0	0
P84098	60S ribosomal protein L19	RPL19	302896,6667	0	0	2	2	13,3	13,3	13,3	5,6693	10611000	6	0	0	0
P35268	60S ribosomal protein L22	RPL22	790483,3333	794963,3333	1,005667419	1	1	10,2	10,2	10,2	2,5405	19025000	6	0	0	0
P83731	60S ribosomal protein L24	RPL24	162773,3333	0	0	1	1	8,3	8,3	8,3	2,8291	4434100	6	0	0	0
P11021	78 kDa glucose-regulated protein	HSPA5	98438,36667	964976,6667	9,80285126	18	17	29,7	29,7	29,7	47,582	95707000	51	0	0	0
P63261	Actin, cytoplasmic 2	ACTG1	3652666,667	9525533,333	2,607829896	22	10	62,4	62,4	40,5	134,02	7,91E+08	77	0	0	0
P63267	Actin, gamma-enteric smooth muscle	ACTG2	275093,3333	0	0	2	2	30,9	9	9	6,6296	18945000	5	0	0	0
P61158	Actin-related protein 3	ACTR3	0	167705	#DIV/0!	3	3	15,3	15,3	15,3	5,3174	7043500	5	0	0	0
P23526	Adenosylhomocysteinase	AHCY	0	82303,66667	#DIV/0!	2	2	6,9	6,9	6,9	5,2467	4938200	4	0	0	0
Q9UKK9	ADP-sugar pyrophosphatase	NUDT5	0	330765	#DIV/0!	4	4	22,8	22,8	22,8	8,7096	7276900	7	0	0	0
P30838	Aldehyde dehydrogenase, dimeric NADP-preferring	ALDH3A1	0	56450,33333	#DIV/0!	2	2	4,6	4,6	4,6	3,8596	3556400	4	0	0	0
A8K2U0	Alpha-2-macroglobulin-like protein 1	A2ML1	0	442193,3333	#DIV/0!	19	19	19,1	19,1	19,1	50,268	90208000	36	0	0	0
O43707	Alpha-actinin-4	ACTN4	0	255700	#DIV/0!	10	10	16,1	16,1	16,1	19,483	42190000	23	0	0	0
P06733	Alpha-enolase	ENO1	725183,3333	3764300	5,190825309	21	21	63,8	63,8	63,8	156,48	2,96E+08	68	0	0	0
P04083	Annexin A1	ANXA1	354506,6667	875946,6667	2,470889123	10	10	30,1	30,1	30,1	28,498	73828000	40	0	0	0
P07355	Annexin A2	ANXA2	2230566,667	10337333,33	4,634397836	27	27	61,9	61,9	61,9	248,51	8,67E+08	115	0	0	0
P09525	Annexin A4	ANXA4	0	279180	#DIV/0!	6	6	26,6	26,6	26,6	11,822	18426000	12	0	0	0
P08758	Annexin A5	ANXA5	0	68933	#DIV/0!	2	2	7,8	7,8	7,8	4,5337	3033000	2	0	0	0
P20073	Annexin A7	ANXA7	0	50733	#DIV/0!	2	2	5,1	5,1	5,1	3,961	2130800	3	0	0	0
P05090	Apolipoprotein D	APOD	296925	1325066,667	4,462630855	7	7	29,6	29,6	29,6	45,048	41121000	11	142	83	58
O75342	Arachidonate 12-lipoxygenase, 12R-type	ALOX12B	72146,5	663720	9,199614673	6	6	11,3	11,3	11,3	19,578	76876000	18	0	0	0
P05089	Arginase-1	ARG1	456906,6667	4083133,333	8,936471344	11	11	43,5	43,5	43,5	37,575	2,32E+08	41	0	0	0
P25705	ATP synthase subunit alpha, mitochondrial	ATP5A1	161363,3333	246046,6667	1,524799108	7	7	17,2	17,2	17,2	17,315	37889000	29	0	0	0
P06576	ATP synthase subunit beta, mitochondrial	ATP5B	343926,6667	193050	0,56131152	8	8	19,7	19,7	19,7	17,524	46717000	22	0	0	0
O15523	ATP-dependent RNA helicase DDX3Y	DDX3Y	0	26156	#DIV/0!	2	2	4,2	4,2	4,2	4,542	3217200	4	0	0	0
P07686	Beta-hexosaminidase subunit beta	HEXB	0	38851,5	#DIV/0!	1	1	3,8	3,8	3,8	2,7901	2253400	2	0	0	0
P21810	Biglycan	BGN	693100	0	0	6	6	26,4	26,4	26,4	15,215	35967000	16	0	0	0
Q13867	Bleomycin hydrolase	BLMH	94813	1176233,333	12,40582339	11	11	33	33	33	34,454	96676000	27	0	164	0
P27482	Calmodulin-like protein 3	CALML3	218850	187823,3333	0,858228619	3	3	30,9	30,9	30,9	7,9575	13420000	9	0	0	0
Q9NZT1	Calmodulin-like protein 5	CALML5	511840	2642300	5,162355424	8	8	60,3	60,3	60,3	49,227	85162000	22	255	0	25
P07384	Calpain-1 catalytic subunit	CAPN1	50404,5	355503,3333	7,053007833	5	5	7,8	7,8	7,8	11,037	42024000	12	0	0	0
Q9UI42	Carboxypeptidase A4	CPA4	0	594426,6667	#DIV/0!	2	2	8,6	8,6	8,6	8,4294	37449000	7	0	0	0
P31944	Caspase-14	CASP14	514723,3333	3052266,667	5,929917043	16	16	52,9	52,9	52,9	83,173	1,61E+08	46	0	0	0
P04040	Catalase	CAT	463266,6667	1857466,667	4,009497769	11	11	30,9	30,9	30,9	52,544	2,02E+08	43	0	0	0
P07858	Cathepsin B	CTSB	0	569180	#DIV/0!	4	4	12,1	12,1	12,1	8,5369	32443000	10	0	0	0
P07339	Cathepsin D	CTSD	98819	1688066,667	17,08240993	8	8	22,6	22,6	22,6	26,251	1,02E+08	27	0	0	0
O60911	Cathepsin L2	CTSV	0	1154133,333	#DIV/0!	6	6	32,9	32,9	32,9	17,841	62324000	19	0	0	0
P60953	Cell division control protein 42 homolog	CDC42	0	82089	#DIV/0!	2	2	16,2	16,2	16,2	3,0159	1477600	2	0	0	0
Q00610	Clathrin heavy chain 1	CLTC	0	14051,6	#DIV/0!	2	2	2,5	2,5	2,5	4,6396	2529300	2	0	0	0
P35606	Coatomer subunit beta	COPB2	0	43873	#DIV/0!	2	2	3	3	3	4,0828	6317800	4	0	0	0
P23528	Cofilin-1	CFL1	0	516530	#DIV/0!	4	4	31,9	31,9	31,9	15,529	15958000	12	0	0	0
P02452	Collagen alpha-1(I) chain	COL1A1	34108,2	0	0	5	5	5,1	5,1	5,1	12,672	7572000	9	0	0	0
P12109	Collagen alpha-1(VI) chain	COL6A1	34556	0	0	2	2	3,2	3,2	3,2	3,1039	5598100	4	0	0	0
P08123	Collagen alpha-2(I) chain	COL1A2	79993,33333	0	0	2	2	1,6	1,6	1,6	13,757	16559000	9	0	0	0
P12111	Collagen alpha-3(VI) chain	COL6A3	3131,85	0	0	2	2	0,7	0,7	0,7	2,6556	1077400	2	0	0	0
O75131	Copine-3	CPNE3	0	145126,6667	#DIV/0!	4	4	12,7	12,7	12,7	7,4679	10449000	5	0	0	0
Q15517	Corneodesmosin	CDSN	526983,3333	619096,6667	1,174793637	5	5	12,3	12,3	12,3	17,003	51573000	12	0	0	0
Q9BYD5	Cornifelin	CNFN	0	1491833,333	#DIV/0!	1	1	19,6	19,6	19,6	5,7829	8951000	5	0	0	0
P35321	Cornifin-A	SPRR1A	0	1628666,667	#DIV/0!	1	1	70,8	19,1	19,1	4,6057	34202000	3	0	0	0
P22528	Cornifin-B	SPRR1B	1225053,333	13414533,33	10,95016271	7	2	70,8	70,8	28,1	18,97	3,07E+08	33	0	0	0
P21291	Cysteine and glycine-rich protein 1	CSRP1	176100	0	0	4	4	36,8	36,8	36,8	7,0714	9605600	6	0	0	0
Q9UGL9	Cysteine-rich C-terminal protein 1	CRCT1	0	3611233,333	#DIV/0!	2	2	50,5	50,5	50,5	8,3217	54168000	9	0	0	0
Q07065	Cytoskeleton-associated protein 4	CKAP4	0	70393,66667	#DIV/0!	6	6	16,4	16,4	16,4	11,894	8024700	8	0	0	0
Q3MJ16	Cytosolic phospholipase A2 epsilon	PLA2G4E	0	32684,5	#DIV/0!	2	2	3,2	3,2	3,2	3,2131	2222600	3	0	0	0
P07585	Decorin	DCN	265049,3333	0	0	4	4	12,8	12,8	12,8	30,961	15108000	7	143	0	193
P81605	Dermcidin	DCD	7795166,667	2541666,667	0,326056745	4	4	35,5	35,5	35,5	15,452	1,55E+08	19	0	0	0
P17661	Desmin	DES	0	43025,66667	#DIV/0!	1	1	8,5	3,2	3,2	3,8583	4130500	4	0	0	0
Q08554	Desmocollin-1	DSC1	892603,3333	1724700	1,932213264	18	18	26,7	26,7	26,7	81,807	3,77E+08	63	0	0	0
Q14574	Desmocollin-3	DSC3	92316,33333	435096,6667	4,713106023	7	7	12,3	12,3	12,3	19,807	77530000	25	0	0	0
Q02413	Desmoglein-1	DSG1	1730266,667	5274566,667	3,048412576	31	31	41,1	41,1	41,1	134,14	8,62E+08	77	0	0	0
P15924	Desmoplakin	DSP	784736,3333	10046766,67	12,80272907	181	181	60,2	60,2	60,2	323,31	5,3E+09	547	0	0	0
P04843	Dolichyl-diphosphooligosaccharide--protein glycosyltransferase subunit 1	RPN1	0	44993,33333	#DIV/0!	3	3	7,7	7,7	7,7	5,2676	4994300	6	0	0	0
P68104	Elongation factor 1-alpha 1	EEF1A1	911357	1498339,667	1,644075446	8	8	29,2	29,2	29,2	39,35	1,52E+08	31	0	137	0
P13639	Elongation factor 2	EEF2	34074,66667	809050	23,74344577	17	17	26,1	26,1	26,1	52,736	1,14E+08	48	0	0	0
Q92817	Envoplakin	EVPL	0	95881	#DIV/0!	9	9	6,3	6,3	6,3	20,615	32791000	19	0	0	0
P58107	Epiplakin	EPPK1	0	41760,33333	#DIV/0!	5	5	8,8	6,2	6,2	19,957	34202000	12	0	0	0
P60842	Eukaryotic initiation factor 4A-I	EIF4A1	0	181316,6667	#DIV/0!	2	2	5,7	5,7	5,7	4,7995	12511000	6	0	0	0
P56537	Eukaryotic translation initiation factor 6	EIF6	325715	972056,3333	2,984376935	3	3	22,9	22,9	22,9	19,729	32109000	10	0	0	0
Q16610	Extracellular matrix protein 1	ECM1	0	960000	#DIV/0!	7	7	20,7	20,7	20,7	20,715	91244000	14	0	167	0
P52907	F-actin-capping protein subunit alpha-1	CAPZA1	0	533670	#DIV/0!	4	4	25,9	25,9	25,9	10,105	21606000	12	0	0	0
P47756	F-actin-capping protein subunit beta	CAPZB	0	260565	#DIV/0!	3	3	13,7	13,7	13,7	4,815	8859200	4	0	0	0
Q16658	Fascin	FSCN1	0	59986,66667	#DIV/0!	1	1	4,9	4,9	4,9	5,0058	5038900	3	0	0	0
P49327	Fatty acid synthase	FASN	0	17483,25	#DIV/0!	3	3	1,9	1,9	1,9	5,2898	4370800	3	0	0	0
Q01469	Fatty acid-binding protein, epidermal	FABP5	2710966,667	15096000	5,568493403	11	11	73,3	73,3	73,3	94,416	4,27E+08	47	0	0	0
Q6ZVX7	F-box only protein 50	NCCRP1	179390	4755366,667	26,50853819	6	6	23,6	23,6	23,6	14,258	1,76E+08	21	0	0	0
P02751	Fibronectin	FN1	34133	0	0	5	5	3	3	3	15,606	10342000	10	0	0	0
P21333	Filamin-A	FLNA	0	22139,83333	#DIV/0!	5	5	3	3	3	11,053	8900200	6	0	0	0
O75369	Filamin-B	FLNB	0	8101,6	#DIV/0!	2	2	1,4	1,4	1,4	4,0473	3451300	3	0	0	0
Q13642	Four and a half LIM domains protein 1	FHL1	64586	0	0	2	2	8,7	8,7	8,7	3,086	3487700	4	0	0	0
P04075	Fructose-bisphosphate aldolase A	ALDOA	300489,3333	1530733,333	5,094135344	12	11	40,9	40,9	33,2	46,796	1,26E+08	36	0	77	0
P09972	Fructose-bisphosphate aldolase C	ALDOC	0	26912	#DIV/0!	1	1	12,1	4,4	4,4	3,2046	1238000	2	0	0	0
P47929	Galectin-7	LGALS7	345058,6667	5281566,667	15,30628608	9	9	65,4	65,4	65,4	91,582	1,69E+08	37	0	0	0
O75223	Gamma-glutamylcyclotransferase	GGCT	488296,6667	2249466,667	4,606762282	9	9	47,3	47,3	47,3	23,357	1,07E+08	31	0	0	0
P17900	Ganglioside GM2 activator	GM2A	0	158060,5	#DIV/0!	2	2	9,8	9,8	9,8	2,7438	2529000	2	0	0	0
Q96QA5	Gasdermin-A	GSDMA	83387,66667	1135813,333	13,62087919	6	6	11,9	11,9	11,9	23,723	87783000	17	0	0	0
P04062	Glucosylceramidase	GBA	0	98282,33333	#DIV/0!	2	2	4,1	4,1	4,1	3,9482	7960900	5	0	0	0
P15104	Glutamine synthetase	GLUL	0	285603,3333	#DIV/0!	4	4	14,2	14,2	14,2	11,398	12852000	10	0	0	0
P09211	Glutathione S-transferase P	GSTP1	149613,3333	1146263,333	7,661505213	7	7	51,9	51,9	51,9	20,117	42763000	15	0	0	0
P04406	Glyceraldehyde-3-phosphate dehydrogenase	GAPDH	3061266,667	11402766,67	3,724852458	16	16	50,4	50,4	50,4	75,31	7,38E+08	52	0	82	0
Q7L5L3	Glycerophosphodiester phosphodiesterase domain-containing protein 3	GDPD3	0	444126,3333	#DIV/0!	5	5	19,2	19,2	19,2	11,714	21318000	9	0	0	0
Q9Y2T3	Guanine deaminase	GDA	0	52317	#DIV/0!	3	3	11,2	11,2	11,2	5,3616	2720500	4	0	0	0
P0DMV9	Heat shock 70 kDa protein 1B	HSPA1B	116134	309576,6667	2,665685042	7	1	19,7	16,5	2,3	15,702	40868000	25	0	0	0
P17066	Heat shock 70 kDa protein 6	HSPA6	0	110653,3333	#DIV/0!	1	1	10,6	1,7	1,7	4,9675	12282000	6	0	0	0
P11142	Heat shock cognate 71 kDa protein	HSPA8	210013,3333	910240	4,334201003	17	14	32,5	32,5	27,4	38,871	1,11E+08	52	0	0	0
P04792	Heat shock protein beta-1	HSPB1	349960	4559766,667	13,02939384	8	8	60	60	60	26,785	2,06E+08	25	0	0	0
P07900	Heat shock protein HSP 90-alpha	HSP90AA1	52886	163570	3,092879023	5	5	16,3	9,2	9,2	15,317	19684000	14	0	0	0
P08238	Heat shock protein HSP 90-beta	HSP90AB1	282463,3333	437700	1,549581657	10	4	17	17	7,9	25,239	71297000	33	0	0	0
P69905	Hemoglobin subunit alpha	HBA1	4599733,333	739016,6667	0,160665111	3	3	56,3	38,7	38,7	9,286	1,28E+08	18	0	0	0
P68871	Hemoglobin subunit beta	HBB	4534133,333	615016,6667	0,135641504	8	4	74,8	61,9	36,1	36,277	1,7E+08	24	0	138	0
P55795	Heterogeneous nuclear ribonucleoprotein H2	HNRNPH2	0	148463,3333	#DIV/0!	1	1	3,8	3,8	3,8	4,8208	7571600	4	0	0	0
Q00839	Heterogeneous nuclear ribonucleoprotein U	HNRNPU	57593	80347,66667	1,395094311	4	4	5,6	5,6	5,6	9,0325	13181000	8	0	0	0
P22626	Heterogeneous nuclear ribonucleoproteins A2/B1	HNRNPA2B1	44774	219650,6667	4,905763762	4	4	14,2	14,2	14,2	6,9854	15865000	10	0	0	0
P42357	Histidine ammonia-lyase	HAL	29437,33333	1248273,667	42,40444107	15	15	33,6	33,6	33,6	58,531	1,3E+08	44	0	0	0
Q99878	Histone H2A type 1-J	HIST1H2AJ	5683366,667	3131133,333	0,55092932	2	1	15,6	15,6	8,6	6,0836	1,32E+08	9	0	0	0
Q99880	Histone H2B type 1-L	HIST1H2BL	5052800	368856,6667	0,073000449	4	4	34,9	34,9	34,9	7,1866	78485000	7	0	0	0
P62805	Histone H4	HIST1H4A	9671300	5552800	0,574152389	9	9	62,1	62,1	62,1	56,291	2,74E+08	34	0	136	0
Q9BYJ1	Hydroperoxide isomerase ALOXE3	ALOXE3	0	157420	#DIV/0!	4	4	9	9	9	19,924	13696000	9	0	0	0
P01876	Ig alpha-1 chain C region	IGHA1	390065	317373,3333	0,813642171	4	4	15	15	15	9,6721	27716000	12	0	0	0
P01857	Ig gamma-1 chain C region	IGHG1	1216233,333	430460	0,353928797	7	4	29,4	29,4	18,8	14,058	74102000	24	0	0	0
P01860	Ig gamma-3 chain C region	IGHG3	176508	0	0	2	1	20,4	11,1	8	3,6196	9962100	4	0	0	0
P01834	Ig kappa chain C region	IGKC	888056,6667	1018153,333	1,146495907	5	5	51,4	51,4	51,4	15,299	28593000	11	0	0	0
O14732	Inositol monophosphatase 2	IMPA2	0	335140	#DIV/0!	2	2	7,3	7,3	7,3	3,8275	13070000	4	0	0	0
P14735	Insulin-degrading enzyme	IDE	0	67509	#DIV/0!	6	6	8,9	8,9	8,9	11,74	10126000	7	0	0	0
Q9NZH8	Interleukin-36 gamma	IL36G	0	94290,45	#DIV/0!	2	2	13	13	13	3,4161	2074400	3	0	0	0
P07476	Involucrin	IVL	0	326440	#DIV/0!	10	10	22,1	22,1	22,1	34,476	32318000	28	0	0	0
P14923	Junction plakoglobin	JUP	1242200	13450333,33	10,82783234	38	38	63,4	63,4	63,4	223,46	2,03E+09	123	152	0	432
Q9UKR3	Kallikrein-13	KLK13	0	158919	#DIV/0!	3	3	13,4	13,4	13,4	5,0345	5721100	6	0	0	0
Q9Y337	Kallikrein-5	KLK5	0	163956,6667	#DIV/0!	2	2	7,5	7,5	7,5	3,5131	6886300	6	0	0	0
P49862	Kallikrein-7	KLK7	0	263036,6667	#DIV/0!	3	3	18,6	18,6	18,6	7,3533	8680200	4	179	0	202
P02788	Lactotransferrin	LTF	850833,3333	64802,66667	0,076163761	16	16	32,8	29,9	29,9	42,644	1,29E+08	55	127	0	280
Q5T7P2	Late cornified envelope protein 1A	LCE1A	0	4261800	#DIV/0!	2	2	61,8	61,8	61,8	9,2167	25571000	10	0	0	0
Q5T7P3	Late cornified envelope protein 1B	LCE1B	0	14256000	#DIV/0!	1	1	66,9	28,8	28,8	3,7533	58942000	3	0	0	0
Q5T752	Late cornified envelope protein 1D	LCE1D	0	3151350	#DIV/0!	1	1	27,2	27,2	27,2	13,586	12605000	2	0	0	0
Q5T753	Late cornified envelope protein 1E	LCE1E	0	17570500	#DIV/0!	2	1	64,4	64,4	26,3	18,815	70282000	8	0	0	0
Q5T754	Late cornified envelope protein 1F	LCE1F	0	8987100	#DIV/0!	2	2	64,4	64,4	64,4	21,63	53924000	5	0	0	0
Q5TA79	Late cornified envelope protein 2A	LCE2A	0	937620	#DIV/0!	1	1	23,6	23,6	23,6	3,9506	5625700	3	0	0	0
Q5TA81	Late cornified envelope protein 2C	LCE2C	1077700	5597700	5,194117101	2	2	23,6	23,6	23,6	13,069	37897000	9	0	0	0
Q5TA82	Late cornified envelope protein 2D	LCE2D	0	1025990	#DIV/0!	1	1	22,7	22,7	22,7	6,7495	6155800	3	0	0	0
Q9BYE3	Late cornified envelope protein 3D	LCE3D	0	1048513,333	#DIV/0!	1	1	37	37	37	3,874	6291000	6	0	0	0
P31025	Lipocalin-1	LCN1	4830866,667	455045	0,094195313	2	2	12,5	12,5	12,5	4,7469	1,23E+08	8	0	0	0
P00338	L-lactate dehydrogenase A chain	LDHA	0	651053,3333	#DIV/0!	4	4	13,6	13,6	13,6	8,5101	35157000	10	0	0	0
P23490	Loricrin	LOR	0	4835600	#DIV/0!	2	2	4,2	4,2	4,2	3,0726	48261000	4	0	0	0
P51884	Lumican	LUM	53691	0	0	2	2	15,1	9,5	9,5	4,8479	1718100	3	0	0	0
P10619	Lysosomal protective protein	CTSA	0	169436,6667	#DIV/0!	1	1	4,2	4,2	4,2	14,212	10289000	4	0	0	0
P11279	Lysosome-associated membrane glycoprotein 1	LAMP1	0	589186,6667	#DIV/0!	2	2	6,2	6,2	6,2	8,4513	33584000	5	0	0	0
P61626	Lysozyme C	LYZ	7012566,667	1298006,667	0,18509723	5	5	43,9	43,9	43,9	21,324	2,24E+08	21	0	0	0
P40121	Macrophage-capping protein	CAPG	0	109300,6667	#DIV/0!	2	2	7,2	7,2	7,2	4,1014	4590500	5	0	0	0
P40925	Malate dehydrogenase, cytoplasmic	MDH1	0	211870	#DIV/0!	2	2	9,6	9,6	9,6	4,5832	9534100	5	0	0	0
P40926	Malate dehydrogenase, mitochondrial	MDH2	0	84699,66667	#DIV/0!	3	3	14,5	14,5	14,5	5,519	5336100	5	0	0	0
P26038	Moesin	MSN	28122,5	37801	1,344155036	4	4	7,1	7,1	7,1	6,4774	4351000	3	0	0	0
P60660	Myosin light polypeptide 6	MYL6	144410	267595	1,853022644	2	2	17,2	17,2	17,2	4,0756	8240100	6	0	0	0
Q7Z406	Myosin-14	MYH14	0	8292,3	#DIV/0!	2	2	2,5	1,2	1,2	3,8381	2562300	4	0	0	0
P35579	Myosin-9	MYH9	0	728580	#DIV/0!	35	33	20,7	20,7	19,4	182,5	2,27E+08	88	0	0	0
P48163	NADP-dependent malic enzyme	ME1	0	21967,66667	#DIV/0!	2	2	3,7	3,7	3,7	3,5713	1845300	4	0	0	0
Q09666	Neuroblast differentiation-associated protein AHNAK	AHNAK	4609,35	29243	6,344278477	14	14	5,8	5,8	5,8	31,798	36452000	28	185	158	1084
P80188	Neutrophil gelatinase-associated lipocalin	LCN2	0	168796,6667	#DIV/0!	3	3	22,2	22,2	22,2	7,4765	4557500	4	0	0	0
P62937	Peptidyl-prolyl cis-trans isomerase A	PPIA	0	417340	#DIV/0!	2	2	9,1	9,1	9,1	3,3387	8346700	2	0	0	0
P23284	Peptidyl-prolyl cis-trans isomerase B	PPIB	210766,6667	125874,6667	0,597222837	3	3	18,5	18,5	18,5	6,7173	12119000	10	0	0	0
O60437	Periplakin	PPL	0	136333,3333	#DIV/0!	16	16	13	13	13	36,706	46217000	34	0	0	0
Q06830	Peroxiredoxin-1	PRDX1	1009650	4082433,333	4,043414385	10	9	45,2	45,2	39,7	24,239	2,29E+08	41	0	0	0
P32119	Peroxiredoxin-2	PRDX2	451490	1881833,333	4,168050972	9	9	34,3	28,8	28,8	20,038	90999000	27	0	0	0
P30041	Peroxiredoxin-6	PRDX6	0	112677	#DIV/0!	2	2	14,7	14,7	14,7	5,0943	3380300	3	0	0	0
P51659	Peroxisomal multifunctional enzyme type 2	HSD17B4	0	40898	#DIV/0!	3	3	5,2	5,2	5,2	5,8472	3435500	4	0	0	0
Q96G03	Phosphoglucomutase-2	PGM2	0	48117	#DIV/0!	3	3	6,7	6,7	6,7	6,1403	3272000	3	0	0	0
P00558	Phosphoglycerate kinase 1	PGK1	77153	444100	5,756095032	5	5	22,3	22,3	22,3	19,938	40138000	16	0	0	0
P18669	Phosphoglycerate mutase 1	PGAM1	0	271323,3333	#DIV/0!	2	2	8,3	8,3	8,3	7,9611	11177000	7	0	0	0
Q6P4A8	Phospholipase B-like 1	PLBD1	0	163656,6667	#DIV/0!	2	2	4,3	4,3	4,3	3,3715	12274000	5	0	0	0
Q13835	Plakophilin-1	PKP1	277296,6667	4112666,667	14,83128779	29	29	49	49	49	143,81	6,06E+08	105	424	0	635
Q15149	Plectin	PLEC	0	17441	#DIV/0!	6	5	1,6	1,6	1,2	10,58	15174000	11	428	0	520
Q15365	Poly(rC)-binding protein 1	PCBP1	0	139976,6667	#DIV/0!	2	2	7	7	7	3,4467	7138800	3	0	0	0
P01833	Polymeric immunoglobulin receptor	PIGR	0	27800,5	#DIV/0!	1	1	2,6	2,6	2,6	3,6751	2279600	2	0	0	0
P02545	Prelamin-A/C	LMNA	629760	1200910,333	1,906933329	25	25	37,2	37,2	37,2	85,595	2,25E+08	82	0	76	0
P12273	Prolactin-inducible protein	PIP	2582233,333	164523,3333	0,063713581	6	6	40,4	40,4	40,4	12,898	74162000	20	0	0	0
P25786	Proteasome subunit alpha type-1	PSMA1	0	152330	#DIV/0!	4	4	20,2	20,2	20,2	6,4925	7768900	6	0	0	0
P25788	Proteasome subunit alpha type-3	PSMA3	0	225070	#DIV/0!	1	1	4,7	4,7	4,7	2,6057	8777700	3	0	0	0
P25789	Proteasome subunit alpha type-4	PSMA4	0	129245	#DIV/0!	2	2	11,1	11,1	11,1	5,7349	3360400	4	0	0	0
P28066	Proteasome subunit alpha type-5	PSMA5	0	272734,3333	#DIV/0!	3	3	14,5	14,5	14,5	6,3908	9000200	6	0	0	0
O14818	Proteasome subunit alpha type-7	PSMA7	99371,33333	328296,6667	3,303736155	4	4	24,6	24,6	24,6	7,6089	17962000	11	0	0	0
P20618	Proteasome subunit beta type-1	PSMB1	0	285856,6667	#DIV/0!	3	3	22,8	22,8	22,8	16,281	12006000	6	0	0	0
P49721	Proteasome subunit beta type-2	PSMB2	0	195933,3333	#DIV/0!	1	1	7,5	7,5	7,5	3,1391	5878100	3	0	0	0
P28070	Proteasome subunit beta type-4	PSMB4	0	236365	#DIV/0!	2	2	15,9	15,9	15,9	5,8838	5200000	3	0	0	0
P28074	Proteasome subunit beta type-5	PSMB5	0	162180,3333	#DIV/0!	5	5	24,7	24,7	24,7	8,6849	7298200	8	0	0	0
P28072	Proteasome subunit beta type-6	PSMB6	0	444015	#DIV/0!	2	2	8,4	8,4	8,4	2,9327	9768400	4	0	0	0
Q8WVV4	Protein POF1B	POF1B	138366,6667	6095933,333	44,05637196	25	25	48,7	48,7	48,7	119,58	5,05E+08	85	0	182	0
P31949	Protein S100-A11	S100A11	0	308816,6667	#DIV/0!	2	2	20	20	20	4,3586	4632200	4	0	0	0
Q9HCY8	Protein S100-A14	S100A14	358280	1462966,667	4,083305422	4	4	58,7	58,7	58,7	16,095	35738000	12	0	0	0
P29034	Protein S100-A2	S100A2	0	1465250	#DIV/0!	3	3	27,6	27,6	27,6	5,085	11722000	4	0	0	0
P31151	Protein S100-A7	S100A7	1578933,333	5179400	3,280315825	8	8	37,6	37,6	37,6	18,612	1,42E+08	23	0	0	0
P05109	Protein S100-A8	S100A8	7703466,667	75526666,67	9,804243977	10	10	46,2	46,2	46,2	116,21	2E+09	38	0	84	0
P06702	Protein S100-A9	S100A9	5526333,333	45226000	8,183726401	10	10	70,2	70,2	70,2	47,247	1,22E+09	40	0	85	0
Q01105	Protein SET	SET	95791	0	0	2	2	8,3	8,3	8,3	3,3678	6513800	4	0	0	0
O43548	Protein-glutamine gamma-glutamyltransferase 5	TGM5	0	230996,6667	#DIV/0!	6	6	13,8	13,8	13,8	16,485	22869000	13	0	0	0
Q08188	Protein-glutamine gamma-glutamyltransferase E	TGM3	198700	2019233,333	10,1622211	21	21	40,7	40,7	40,7	56,986	2,66E+08	71	0	154	0
P22735	Protein-glutamine gamma-glutamyltransferase K	TGM1	168759,3333	2140966,667	12,6865082	20	20	36	36	36	91,868	3,26E+08	48	397	110	234
P00491	Purine nucleoside phosphorylase	PNP	0	125516,3	#DIV/0!	2	2	11,1	11,1	11,1	3,753	6777900	4	0	0	0
P55786	Puromycin-sensitive aminopeptidase	NPEPPS	0	29524	#DIV/0!	3	3	5,4	5,4	5,4	5,5676	2834300	3	0	0	0
P14618	Pyruvate kinase PKM	PKM	183196,6667	1829033,333	9,983988064	19	19	47,3	47,3	47,3	81,365	1,99E+08	53	151	88	199
P50395	Rab GDP dissociation inhibitor beta	GDI2	0	81140,33333	#DIV/0!	2	2	6,1	6,1	6,1	4,9009	6815700	4	0	0	0
P61106	Ras-related protein Rab-14	RAB14	0	59692,5	#DIV/0!	1	1	7	7	7	3,6394	1910200	2	0	0	0
P51149	Ras-related protein Rab-7a	RAB7A	0	100383	#DIV/0!	2	2	12,1	12,1	12,1	4,3743	2810800	3	0	0	0
P02753	Retinol-binding protein 4	RBP4	246885	0	0	1	1	5	5	5	2,7156	5807300	4	0	0	0
Q53RT3	Retroviral-like aspartic protease 1	ASPRV1	0	2425066,667	#DIV/0!	3	3	13,7	13,7	13,7	18,292	80027000	11	0	0	0
Q9H1E1	Ribonuclease 7	RNASE7	0	396313,3333	#DIV/0!	3	3	32,7	32,7	32,7	12,727	8322600	6	0	0	0
P13489	Ribonuclease inhibitor	RNH1	0	32162	#DIV/0!	2	2	7,6	7,6	7,6	3,4581	2508600	6	0	0	0
P02787	Serotransferrin	TF	516593,3333	122410	0,236956213	13	13	25,5	22,6	22,6	27,341	73579000	36	0	0	0
Q96P63	Serpin B12	SERPINB12	942880	3606933,333	3,825442616	13	13	44,4	44,4	44,4	117,2	2,73E+08	51	0	186	0
P29508	Serpin B3	SERPINB3	331770	4891100	14,74244205	14	7	42,3	42,3	24,4	79,621	3,45E+08	53	0	0	0
P48594	Serpin B4	SERPINB4	0	150855	#DIV/0!	2	2	25,6	7,7	7,7	3,6644	6335800	3	0	0	0
P36952	Serpin B5	SERPINB5	0	169955,3333	#DIV/0!	3	3	12,8	12,8	12,8	7,2195	11217000	7	0	0	0
P50452	Serpin B8	SERPINB8	0	59989,66667	#DIV/0!	2	2	7,8	7,8	7,8	5,1621	3779400	5	0	0	0
Q8NEX9	Short-chain dehydrogenase/reductase family 9C member 7	SDR9C7	0	195948,3	#DIV/0!	4	4	16,9	16,9	16,9	8,446	11169000	7	0	0	0
Q9Y3R4	Sialidase-2	NEU2	0	157543	#DIV/0!	3	3	11,3	11,3	11,3	6,646	8507400	5	0	0	0
P84550	SKI family transcriptional corepressor 1	SKOR1	0	36390,5	#DIV/0!	1	1	1,1	1,1	1,1	2,6412	3420700	3	0	0	0
Q5T750	Skin-specific protein 32	XP32	1882646,667	12490000	6,634277276	9	9	48,8	48,8	48,8	28,15	4,74E+08	30	195	0	27
P35325	Small proline-rich protein 2B	SPRR2B	3079566,667	19152333,33	6,219165034	7	0	79,2	79,2	0	35,051	4E+08	43	0	0	0
P22532	Small proline-rich protein 2D	SPRR2D	0	1609033,333	#DIV/0!	1	1	79,2	25	25	2,811	28963000	4	0	0	0
P22531	Small proline-rich protein 2E	SPRR2E	0	2237933,333	#DIV/0!	1	1	79,2	18,1	18,1	2,637	42461000	5	0	0	0
Q9BYE4	Small proline-rich protein 2G	SPRR2G	465510	0	0	1	1	61,6	31,5	31,5	4,5825	4655100	2	0	0	0
Q96PI1	Small proline-rich protein 4	SPRR4	0	3227933,333	#DIV/0!	4	4	43	43	43	8,1485	77471000	10	0	0	0
Q9NY59	Sphingomyelin phosphodiesterase 3	SMPD3	0	29197	#DIV/0!	2	2	6,6	6,6	6,6	3,7197	1693400	3	0	0	0
P38646	Stress-70 protein, mitochondrial	HSPA9	0	41330	#DIV/0!	2	2	4,4	4,4	4,4	4,0004	3141100	2	0	0	0
P00441	Superoxide dismutase [Cu-Zn]	SOD1	0	282804	#DIV/0!	3	3	29,2	29,2	29,2	6,8339	7840800	5	0	0	0
Q6UWP8	Suprabasin	SBSN	231970	543186,6667	2,341624635	5	5	22	22	22	73,799	74415000	32	0	0	0
P50990	T-complex protein 1 subunit theta	CCT8	0	26066	#DIV/0!	3	3	7,1	7,1	7,1	4,6092	1928900	5	0	0	0
P10599	Thioredoxin	TXN	606195	3756000	6,196026031	5	5	31,4	31,4	31,4	12,005	87363000	18	0	0	0
Q9BQ50	Three prime repair exonuclease 2	TREX2	0	385886,6667	#DIV/0!	3	3	18,6	18,6	18,6	6,7891	13892000	9	0	0	0
P19971	Thymidine phosphorylase	TYMP	0	50477,33333	#DIV/0!	2	2	5,8	5,8	5,8	4,2405	3634400	6	0	0	0
P37837	Transaldolase	TALDO1	0	117649	#DIV/0!	2	2	7,7	7,7	7,7	3,4337	6000000	5	0	0	0
P55072	Transitional endoplasmic reticulum ATPase	VCP	0	383903,3333	#DIV/0!	14	14	26,7	26,7	26,7	32,771	51827000	31	0	0	0
P29401	Transketolase	TKT	0	250480	#DIV/0!	3	3	8,5	8,5	8,5	8,5964	19233000	9	0	0	0
P51571	Translocon-associated protein subunit delta	SSR4	0	358883,3333	#DIV/0!	2	2	17,3	17,3	17,3	4,9228	7536500	5	0	0	0
P49755	Transmembrane emp24 domain-containing protein 10	TMED10	0	278880	#DIV/0!	3	3	20,5	20,5	20,5	5,6769	10040000	6	0	0	0
Q9BVK6	Transmembrane emp24 domain-containing protein 9	TMED9	0	136257,6667	#DIV/0!	2	2	14,5	14,5	14,5	4,7825	5722800	5	0	0	0
P60174	Triosephosphate isomerase	TPI1	94687,5	550613,3333	5,815058306	7	7	44,6	44,6	44,6	19,786	31301000	21	0	0	0
Q71U36	Tubulin alpha-1A chain	TUBA1A	833583,3333	2254833,333	2,704988503	9	0	31,5	31,5	0	34,449	1,95E+08	35	0	0	0
Q9BQE3	Tubulin alpha-1C chain	TUBA1C	0	77750,66667	#DIV/0!	1	1	33,2	5,3	5,3	3,6128	4898300	4	0	0	0
P68371	Tubulin beta-4B chain	TUBB4B	128666,6667	324526,6667	2,522227979	7	2	17,8	17,8	6,1	15,301	27192000	18	0	0	0
Q5VVQ6	Ubiquitin thioesterase OTU1	YOD1	0	389106,6667	#DIV/0!	5	5	20,1	20,1	20,1	15,041	18677000	14	0	0	0
P62987	Ubiquitin-60S ribosomal protein L40	UBA52	3637766,667	5046900	1,387362209	5	5	38,3	38,3	38,3	39,914	1,56E+08	18	0	0	0
P08670	Vimentin	VIM	2658900	368933,3333	0,138754121	27	24	59,9	57,9	52,6	88,654	3,09E+08	82	0	0	0
P18206	Vinculin	VCL	16672,5	185483,3333	11,12510621	9	9	10,9	10,9	10,9	23,418	44234000	22	0	0	0
P38606	V-type proton ATPase catalytic subunit A	ATP6V1A	0	114554,6667	#DIV/0!	4	4	11,8	11,8	11,8	10,688	11685000	7	0	0	0
P21281	V-type proton ATPase subunit B, brain isoform	ATP6V1B2	0	147260	#DIV/0!	2	2	5,3	5,3	5,3	3,4364	11044000	5	0	0	0
P25311	Zinc-alpha-2-glycoprotein	AZGP1	609306,6667	843743,3333	1,384759727	10	10	39,3	39,3	39,3	21,945	78465000	38	0	0	0
A0A1B0GTR4			0	810820	#DIV/0!	2	2	15,7	15,7	15,7	4,3292	9729900	6	0	0	0
P0DP25			344630	0	0	1	1	11,4	11,4	11,4	3,6832	9305100	3	0	0	0
S4R460		IGHV3OR16-9	144886,6667	0	0	1	1	19,8	19,8	19,8	4,1274	4648700	5	0	0	0

Venn Diagram in K562 EVs		
Shared between EV CTR and SAHA	P09211	GSTP1
	P35268	RPL22
	P13639	EEF2
	Q13867	BLMH
	P62854	RPS26
	P14618	PKM
	P31944	CASP14
	P68104	EEF1A1
	P10599	TXN
	P47929	LGALS7B
	P23396	RPS3
	O75342	ALOX12B
	P08238	HSP90AB1
	Q01469	FABP5
	P32119	PRDX2
	P12273	PIP
	P11142	HSPA8
	P63104	YWHAZ
	P22626	HNRNPA2B1
	P01876	
	P04040	CAT
	Q09666	AHNAK
	P62258	YWHAE
	P63261	ACTG1
	P07339	CTSD
	P35325	SPRR2B
	Q9HCY8	S100A14
	Q5T750	C1orf68
	P05089	ARG1
	P00558	PGK1
	P62857	RPS28
	P04083	ANXA1
	P62249	RPS16
	P31025	LCN1
	Q99878	HIST1H2AJ
	Q06830	PRDX1
	Q6UWP8	SBSN
	O75223	GGCT
	Q71U36	TUBA1A
	P01834	
	P05090	APOD
	P61626	LYZ
	P60660	MYL6
	P0DMV9	HSPA1B
	Q96QA5	GSDMA
	Q14574	DSC3
	P27482	CALML3
	P08670	VIM
	P01857	
	Q96P63	SERPINB12
	P56537	EIF6
	P29508	SERPINB3
	P81605	DCD
	Q99880	HIST1H2BL
	P31151	S100A7
	Q6ZVX7	NCCRP1
	P68871	HBB
	Q5TA81	LCE2C
	P04075	ALDOA
	P60174	TPI1
	P31947	SFN
	P02545	LMNA
	P25705	ATP5A1
	Q08554	DSC1
	P22735	TGM1
	P06733	ENO1
	Q15517	CDSN
	P62987	UBA52
	P11021	HSPA5
	P07384	CAPN1
	P25311	AZGP1
	P02787	TF
	P06702	S100A9
	P62805	HIST1H4F
	P07355	ANXA2
	P23284	PPIB
	O14818	PSMA7
	Q13835	PKP1
	Q08188	TGM3
	P18206	VCL
	Q02413	DSG1
	P42357	HAL
	P08865	RPSA
	P02788	LTF
	P62913	RPL11
	P04406	GAPDH
	P04792	HSPB1
	P14923	JUP
	P15924	DSP
	P68371	TUBB4B
	Q00839	HNRNPU
	Q8WVV4	POF1B
	P69905	HBA2
	P07900	HSP90AA1
	P26373	RPL13
	Q9NZT1	CALML5
	P22528	SPRR1B
	P05109	S100A8
	P26038	MSN
	P06576	ATP5B
Unique in EV SAHA	P61981	YWHAG
	P61247	RPS3A
	P62081	RPS7
	P49189	ALDH9A1
	P10809	HSPD1
	P27635	RPL10
	P30050	RPL12
	P61158	ACTR3
	P23526	AHCY
	Q9UKK9	NUDT5
	P30838	ALDH3A1
	A8K2U0	A2ML1
	O43707	ACTN4
	P09525	ANXA4
	P08758	ANXA5
	P20073	ANXA7
	O15523	
	P07686	HEXB
	Q9UI42	CPA4
	P07858	CTSB
	O60911	CTSV
	P60953	CDC42
	Q00610	CLTC
	P35606	COPB2
	P23528	CFL1
	O75131	CPNE3
	Q9BYD5	CNFN
	P35321	SPRR1A
	Q9UGL9	CRCT1
	Q07065	CKAP4
	Q3MJ16	PLA2G4E
	P17661	DES
	P04843	RPN1
	Q92817	EVPL
	P58107	EPPK1
	P60842	EIF4A1
	Q16610	ECM1
	P52907	CAPZA1
	P47756	CAPZB
	Q16658	FSCN1
	P49327	FASN
	P21333	FLNA
	O75369	FLNB
	P09972	ALDOC
	P17900	GM2A
	P04062	GBA
	P15104	GLUL
	Q7L5L3	GDPD3
	Q9Y2T3	GDA
	P17066	HSPA6
	P55795	HNRNPH2
	Q9BYJ1	ALOXE3
	O14732	IMPA2
	P14735	IDE
	Q9NZH8	IL36G
	P07476	IVL
	Q9UKR3	KLK13
	Q9Y337	KLK5
	P49862	KLK7
	Q5T7P2	LCE1A
	Q5T7P3	LCE1B
	Q5T752	LCE1D
	Q5T753	LCE1E
	Q5T754	LCE1F
	Q5TA79	LCE2A
	Q5TA82	LCE2D
	Q9BYE3	LCE3D
	P00338	LDHA
	P23490	LOR
	P10619	CTSA
	P11279	LAMP1
	P40121	CAPG
	P40925	MDH1
	P40926	MDH2
	Q7Z406	MYH14
	P35579	MYH9
	P48163	ME1
	P80188	LCN2
	P62937	PPIA
	O60437	PPL
	P30041	PRDX6
	P51659	HSD17B4
	Q96G03	PGM2
	P18669	PGAM1
	Q6P4A8	PLBD1
	Q15149	PLEC
	Q15365	PCBP1
	P01833	PIGR
	P25786	PSMA1
	P25788	PSMA3
	P25789	PSMA4
	P28066	PSMA5
	P20618	PSMB1
	P49721	PSMB2
	P28070	PSMB4
	P28074	PSMB5
	P28072	PSMB6
	P31949	S100A11
	P29034	S100A2
	O43548	TGM5
	P00491	PNP
	P55786	NPEPPS
	P50395	GDI2
	P61106	RAB14
	P51149	RAB7A
	Q53RT3	ASPRV1
	Q9H1E1	RNASE7
	P13489	RNH1
	P48594	SERPINB4
	P36952	SERPINB5
	P50452	SERPINB8
	Q8NEX9	SDR9C7
	Q9Y3R4	NEU2
	P84550	SKOR1
	P22532	SPRR2D
	P22531	SPRR2E
	Q96PI1	SPRR4
	Q9NY59	SMPD3
	P38646	HSPA9
	P00441	SOD1
	P50990	CCT8
	Q9BQ50	TREX2
	P19971	TYMP
	P37837	TALDO1
	P55072	VCP
	P29401	TKT
	P51571	SSR4
	P49755	TMED10
	Q9BVK6	TMED9
	Q9BQE3	TUBA1C
	Q5VVQ6	YOD1
	P38606	ATP6V1A
	P21281	ATP6V1B2
	A0A1B0GTR4	NEU2
Unique in EV CTRL	P12109	COL6A1
	P01860	
	S4R460	ENSP00000474135
	P02751	FN1
	P63267	ACTG2
	P08123	COL1A2
	Q9BYE4	SPRR2G
	P62701	RPS4X
	P21291	CSRP1
	P51884	LUM
	P02452	COL1A1
	P21810	BGN
	Q01105	SET
	P62263	RPS14
	P07585	DCN
	P12111	COL6A3
	P0DP25	CALM3
	P18621	RPL17
	Q13642	FHL1
	P62277	RPS13
	P84098	RPL19
	P62269	RPS18
	P02753	RBP4
	P83731	RPL24

**Table 4:** Downstream analysis by IPA tool—K562 EVs

Function	p-value	Activation state	Activation z-score	Molecules (n)
Apoptosis of tumor cell lines	3.22E-11	–	− 1.274	58
Cell death of cancer cells	3.48E-16	–	− 1.059	33
Interaction of tumor cell lines	1.68E-06	–	1.006	22
Cell movement of granulocytes	4.19E-07	–	0.107	22
Transmigration of leukocytes	3.33E-07	–	0.239	13
Cell movement of lymphocytes	2.00E-06	–	0.639	22
Cell movement of phagocytes	3.47E-07	–	0.737	29
Leukocyte migration	1.06E-11	–	1.023	49
Cell movement of leukocytes	1.87E-11	–	1.109	44
Cell movement of myeloid cells	6.99E-08	–	1.131	30
Migration of myeloid cells	1.43E-06	–	− 0.155	14
Immune response of cells	9.66E-08	–	0.484	30
Inflammation of body cavity	2.41E-11	–	− 0.228	51
Inflammation of organ	3.52E-28	–	− 0.875	91
Development of benign tumor	8.37E-07	–	− 0.404	28
Development of malignant tumor	2.96E-07	–	− 0.351	182
Hematopoietic neoplasm	1.72E-09	–	− 0.017	103
Invasion of tumor	2.20E-07	–	0.078	16
Formation of solid tumor	8.55E-09	–	0.397	209
Metastatic solid tumor	1.62E-08	–	0.18	33
Hematological lymphatic system tumor	1.29E-09	–	0.201	104
Tumorigenesis of tissue	1.18E-15	–	0.23	241
Invasive tumor	1.78E-10	–	1.354	55
Metastasis	1.02E-09	–	1.354	48
Vasculogenesis	2.23E-07	–	1.862	36
Angiogenesis	1.10E-09	Increased	2.112	46
Development of vasculature	4.13E-09	Increased	2.114	48
Cell proliferation of tumor cell lines	5.46E-10	Increased	2.538	71
Migration of cells	1.28E-15	Increased	2.552	90
Cell movement of tumor cell lines	3.83E-11	Increased	2.832	53
Cell movement	1.23E-16	Increased	2.951	98
Migration of tumor cell lines	2.31E-10	Increased	3.409	47
Invasion of tumor cell lines	5.75E-09	Increased	3.648	43
Cell survival	5.04E-17	Increased	3.724	77
Cell viability	7.58E-14	Increased	3.812	69

Most relevant biological functions predicted to be modulated by K562 EVs (SAHA vs CTR) in receiving cells. z-score £ −2 indicates decreased function activation induced by EV SAHA compared to EV CTR. z-score ^3^ 2 indicated increased function activation induced by EV SAHA compared to EV CTR

These observations suggest the potential therapeutic value of SAHA in particular when combined with complementary therapies.

### SAHA-inducedEVs affect tumorigenicity but not resistance to SAHA in leukemic cells

Following molecular assessments and in silico predictions, we interrogated the functional impact of EV SAHA on recipient leukemic cells. Specifically, we evaluated whether EV SAHA influences tumorigenicity and modulates resistance to SAHA treatment.

**Fig. 4:**
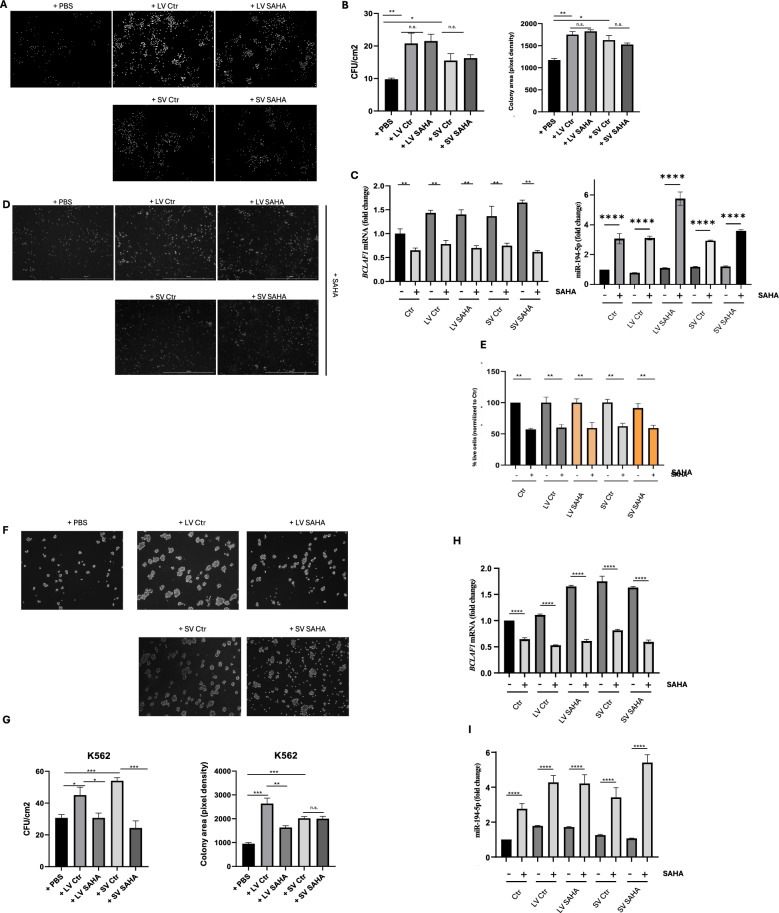
Tumorigenic capability, drug resistance and molecular delivery induced by EV SAHA in U937 and K562. **A** Images acquired with Cytation of U937 colony formation upon treatment with PBS, LV Ctr, LV SAHA, SV Ctr, and SV SAHA; **B** *Left, n*umber of newly formed colonies (per cm^2^); *Right,* area of the colonies (indicated as pixel density). Error bars indicate standard deviation; **C** *Left*, *BCLAF1*mRNA yield in U937 cells exposed to EVs from different sources and subsequently untreated or treated with SAHA (5 µM at 24 h), normalized to GAPDH expression; *Right*, miR-194-5p yield in U937 cells exposed to EVs from different sources and subsequently untreated or treated with SAHA (5 µM at 24 h), normalized to RNU6 expression; **D** Survival and tumorigenic capability of U937 cells pretreated with EVs from different sources followed by SAHA treatment (5 µM at 24 h). Images acquired with Agilent BioTek Cytation 5; **E** Propidium Iodide-based evaluation of cell survival following SAHA treatment induced by EVs from different sources. Error bars indicate standard deviation; **F** Images acquired with Cytation of K562 colony formation upon treatment with PBS, LV Ctr, LV SAHA, SV Ctr, and SV SAHA; **G** *Left, n*umber of newly formed colonies (per cm^2^); *Right,* area of the colonies (indicated as pixel density). Error bars indicate standard deviation; **H** *BCLAF1*mRNA yield in K562 cells exposed to EVs from different sources and subsequently untreated or treated with SAHA (5 µM at 24 h), normalized to GAPDH expression; **I** miR-194-5p yield in K562 cellsexposed to EVs from different sources and subsequently untreated or treated with SAHA (5 µM at 24 h), normalized to *RNU6* expression

In U937 cells, neither EV Ctr nor EV SAHA significantly affected cell viability or cell cycle (Fig. S3A-B).In a tumorigenicity assay, however, AML-derived EV SAHA elicited pro-tumoral effects comparable to those observed with EV Ctr (Fig. 4A, B). Notably, EV treatments increased *BCLAF1*mRNA levels in recipient cells, gain that was abrogated by SAHA treatment (Fig. 4C Left). Expectedly, the EV intervention did not enhance the miRNA levels in recipient AML cells; instead, SAHA induced miR-194-5p overexpression regardless of EV exposure (Fig. 4C Right). Furthermore, neither EV Ctr nor EV SAHA conferred resistance to the treatment in recipient cells (Fig. 4D, E).

Comparable evaluations were performed in K562 cells (Fig. 4). While EVs did not affect viability or cell cycle (Fig. S3C-D), EV SAHA (both SV and LV) produced fewer new tumor colonies relative to EV Ctr (Fig. 4F, G), indicating an anti-neoplastic effect. Consistent with the AML findings, EV treatments delivered *BCLAF1* mRNA to recipient CML cells, with only LV Ctr showing a restrained *BCLAF1*accumulation (Fig. 4H). Regarding the miRNA expression, no significant differences were observed between cells treated with EV SAHA and those receiving EV Ctr, although LVs appeared to deliver greater amounts of miR-194-5p than SVs. Interestingly, EV exposure prior SAHA treatment resulted in an overall enhancement of miR-194-5p expression compared to SAHA treatment alone (Fig. 4I).

In summary, our findings suggest that EV SAHA does not exacerbate tumor progression in AML, whereas it may inhibit malignant development in CML. These results underscore the context-dependent therapeutic potential of SAHA in leukemia.

## Discussion

EV-mediated communication between cancer cells and their microenvironment is gaining increasing recognition as a pivotal process in tumor progression, as it significantly contributes to numerous cancer-related processes, such as tumorigenesis, tumor growth, angiogenesis, immune escape, metastasis, and drug resistance [45, 46]. However, albeit their relevance, the impact of anti-cancer treatments on vesicle-mediated communication is often underestimated and therefore under explored.

This study provides valuable insights into the role of SAHA in modulating EV secretion and composition, while also offering interesting speculations on the potential function of SAHA-modified EVs in Leukemia. By altering both the quantity and molecular content of EVs, SAHA affects intracellular dynamics and extracellular communication, presenting new avenues for therapeutic exploration.

Our previous efforts revealed that SAHA reverses the miR-194-5p/BCLAF1 imbalance responsible for the differentiation arrest and apoptosis resistance characteristic of Leukemia cells. In this study, we uncover that SAHA, while upregulating miR-194-5p expression at the expense of BCLAF1 intracellularly, it selectively fosters BCLAF1 (at both mRNA and protein level) loading into EVs and reduces the miRNA levels in the same compartment. This highlights a previously unrecognized role for SAHA in regulating selective cargo sorting into vesicles, potentially reshaping the tumor microenvironment. Regardless of whether this is traceable to waste disposal dynamics or communication means, EV-associated content may influence recipient cells and considering the anti-apoptotic role of BCLAF1, we speculated whether EV-BCLAF1 endows cancer cells with a phenotype refractory to SAHA. Noteworthy, the overall increase in extracellular BCLAF1 did not confer recipient cancer cells with resistance to SAHA treatment. To explain this result, we initially hypothesized a reduction in EV uptake yet the *BCLAF1*mRNA levels are increased in AML cells treated with EV SAHA, suggesting that the vesicles are internalized by recipient cells and avoid lysosome degradation. Interestingly, treatment with SAHA impaired even *BCLAF1*gained via EV communication. This effect may result from SAHA’s dual role, whereby it enhances miR-194-5p expression while simultaneously limiting its incorporation into EVs, leading to elevated intracellular miRNA levels which could further restrict the accumulation of BCLAF1.

In addition to our molecules of interest, SAHA significantly modulated the overall miRNA profile and proteomic landscape of Leukemia-derived EVs. Such altered vesicular composition was predicted—and confirmed for some biological functions—not to exacerbate the aggressive phenotype of recipient cancer cells, as demonstrated by our functional analysis. Moreover, SAHA modulated the loading of many factors linked to drug resistance and sensitivity, underscoring a dual role for SAHA who has the potential to limit tumor progression while enhancing therapeutic responsiveness, thereby suggesting new options for potential combinatorial therapies.

Trials exploring the use of SAHA in combination with other drugs are ongoing (NCT00392353, NCT01522976) or concluded (NCT00948064, NCT00875745, NCT00479232, NCT00275080, NCT01550224, NCT01534260), and, despite showing promising results in some early-phase studies, the overall success of these trials varies. Our findings suggest the possibility of testing SAHA in combination with drugs for which trials have not been designed yet. Moreover, previous trials investigated SAHA in combination with other therapeutics, while our results suggest adopting a sequential approach, wherein SAHA is used prior to other drugs. In this regard, pre-treatment with SAHA might directly kill malignant cells meanwhile increasing the efficacy of cisplatin and paclitaxel, due to impaired delivery of actin and HSP90 as well as increased transportation of miR-424-5p via EV SAHA. Similarly, these vesicles have the potential to increase the sensitivity to doxorubicin, because of the unique presence of Cul3 in EV SAHA. Moreover, the ability of SAHA to enhance sensitivity to other treatments could allow for dose reductions, potentially minimizing toxicity-related side effects—one of the leading causes of treatment-associated mortality [47]. Additionally, SAHA pre-treatment may influence the efficacy of cell-based and antibody-based therapies, for instance by upregulating surface antigen expression or attenuating immunosuppressive signaling, thereby increasing tumor susceptibility to both the host immune system and CAR-T cells [48, 49].

These findings highlight SAHA’s potential to modulate the extracellular compartment as part of a broader anti-cancer strategy thereby supporting its potential clinical relevance as a component of combination therapies aimed at overcoming resistance in Leukemia.

Importantly, these predictions were only observed in AML cells, as EV SAHA from CML cells were predicted to enhance some tumor-supportive function. SAHA-induced EVs demonstrated a context-dependent impact also at functional level. In CML, EV SAHA treatment hindered the process of colony formation, while no differences between EV Ctr and EV SAHA were observed in AML cells. These different outcomes may be attributed to the diverse differentiation potential, genetic, epigenetic and molecular background, as well as cell lineage and function of acute versus chronic leukemia cells [50]. Moreover, SAHA also exerted differential effects on EV secretion in AML and CML cells, likely reflecting cell-specific differences invesicle biogenesis pathways or sensitivity to SAHA-induced stress, therefore emphasizing the importance of tailoring therapeutic approaches to specific Leukemia subtype.

In conclusion, SAHA’s effects on leukemic EV secretion and molecular composition, along with its direct cytotoxicity toward tumor cells, support its application as a therapeutic agent, especially in combination regimens customized to specific leukemic subtypes. Our findings provide a speculative yet promising foundation for future research into novel therapeutic strategies, encouraging further investigation into the mechanisms underlying these effects and their potential clinical implications.

## Supplementary Information

Additional file 1

Additional file 2

Additional file 3

Additional file 4

## Data Availability

The proteomics data have been deposited to the ProteomeXchange Consortium via the PRIDE partner repository with the dataset identifier PXD042168. All data generated during and/or analyzed during the current study are available upon reasonable request from the corresponding authors.

## Abbreviations

ACTG1: Actin
AML: Acute myeloid leukemia
BCLAF1: Bcl-2-associated transcription factor 1
CML: Chronic myeloid leukemia
Cul3: Cullin-3
EVs: Extracellular vesicles
HSP90: Heat shock protein 90
ILF2: Interleukin enhancer binding factor 2
LC–MS/MS: Tandem mass spectrometry
LVs: Large vesicles
SAHA: Vorinostat
SVs: Small vesicles
