# Supplementary material for: Vorinostat impairs the cancer-driving potential of leukemia-secreted extracellular vesicles
Source: J Transl Med. 2025 Apr 10;23:421. doi: 10.1186/s12967-025-06361-1 (PMC11987450; doi:10.1186/s12967-025-06361-1)
Supplement: Supplementary file 1 — Additional file 1 [file 12967_2025_6361_MOESM1_ESM.docx]

**Supplementary Table 1 miRNA differentially expressed in U937 EVs upon SAHA treatment**

|  |  | **miRNAs** | **2^(-ΔΔCt)** |
| --- | --- | --- | --- |
| Decreased in EV SAHA |  | hsa-miR-200b-5p | 0,00029661 |
|  |  | U6 snRNA | 0,00319943 |
|  |  | hsa-miR-188-3p | 0,00398687 |
|  |  | hsa-miR-183-5p | 0,00697867 |
|  |  | hsa-let-7f-5p | 0,00711915 |
|  |  | hsa-miR-24-2-5p | 0,01954288 |
|  |  | hsa-miR-331-3p | 0,02675307 |
|  |  | hsa-miR-665 | 0,02734278 |
|  |  | hsa-miR-455-3p | 0,02838206 |
|  |  | hsa-let-7d-5p | 0,02844266 |
|  |  | hsa-miR-1538 | 0,02853371 |
|  |  | SNORD38B | 0,02977056 |
|  |  | hsa-miR-103a-3p | 0,03180279 |
|  |  | hsa-miR-96-5p | 0,03893361 |
|  |  | hsa-miR-107 | 0,03951938 |
|  |  | hsa-miR-17-3p | 0,04016278 |
|  |  | hsa-miR-15b-5p | 0,04068365 |
|  |  | hsa-miR-664a-3p | 0,04479095 |
|  |  | hsa-miR-182-5p | 0,05019443 |
|  |  | hsa-miR-9-5p | 0,05165887 |
|  |  | hsa-let-7c-5p | 0,05186561 |
|  |  | hsa-miR-30c-5p | 0,05194227 |
|  |  | hsa-miR-374b-5p | 0,05436583 |
|  |  | hsa-let-7a-5p | 0,05693707 |
|  |  | hsa-miR-450a-5p | 0,05892285 |
|  |  | hsa-miR-142-3p | 0,0637888 |
|  |  | hsa-miR-199b-5p | 0,06597042 |
|  |  | hsa-miR-181a-2-3p | 0,06977171 |
|  |  | hsa-miR-7-5p | 0,0707048 |
|  |  | hsa-miR-671-5p | 0,07787192 |
|  |  | hsa-miR-15a-5p | 0,08194899 |
|  |  | hsa-miR-744-5p | 0,08234655 |
|  |  | hsa-miR-339-5p | 0,08264607 |
|  |  | hsa-miR-26a-5p | 0,08368118 |
|  |  | hsa-miR-1227-3p | 0,08461387 |
|  |  | hsa-let-7g-5p | 0,08701724 |
|  |  | hsa-miR-324-3p | 0,08748157 |
|  |  | hsa-miR-29a-5p | 0,0903543 |
|  |  | hsa-miR-106b-5p | 0,09180802 |
|  |  | hsa-miR-29b-3p | 0,09235923 |
|  |  | hsa-miR-20a-3p | 0,09308987 |
|  |  | hsa-miR-501-5p | 0,09425892 |
|  |  | hsa-miR-2113 | 0,0972835 |
|  |  | hsa-miR-145-5p | 0,09785613 |
|  |  | hsa-miR-28-5p | 0,10063737 |
|  |  | hsa-miR-146b-5p | 0,10163491 |
|  |  | hsa-miR-1972 | 0,10173937 |
|  |  | hsa-miR-33a-5p | 0,10238342 |
|  |  | hsa-miR-652-3p | 0,10466435 |
|  |  | hsa-miR-532-3p | 0,10983738 |
|  |  | hsa-miR-454-3p | 0,11209478 |
|  |  | hsa-miR-20a-5p | 0,11549009 |
|  |  | hsa-miR-181a-5p | 0,12412125 |
|  |  | hsa-miR-18b-5p | 153,925132 |
|  |  | hsa-miR-30b-5p | 0,12678869 |
|  |  | hsa-miR-1207-5p | 0,12863098 |
|  |  | hsa-miR-340-5p | 0,13074014 |
|  |  | hsa-miR-26b-5p | 0,13539177 |
|  |  | hsa-miR-2110 | 0,13570145 |
|  |  | hsa-miR-155-5p | 0,13853006 |
|  |  | hsa-miR-32-5p | 0,13873955 |
|  |  | hsa-miR-196b-5p | 0,14313248 |
|  |  | hsa-miR-1237-3p | 0,14394055 |
|  |  | hsa-miR-320c | 0,14423058 |
|  |  | hsa-miR-374a-5p | 0,14908748 |
|  |  | hsa-miR-1249 | 0,14987992 |
|  |  | hsa-miR-501-3p | 0,1501688 |
|  |  | hsa-miR-29a-3p | 0,15309149 |
|  |  | hsa-miR-130b-3p | 0,15432279 |
|  |  | hsa-miR-122-5p | 0,15753289 |
|  |  | hsa-miR-22-3p | 0,16218587 |
|  |  | hsa-miR-17-5p | 0,16518241 |
|  |  | hsa-miR-365a-3p | 0,16528045 |
|  |  | hsa-miR-431-5p | 0,16976136 |
|  |  | hsa-miR-194-5p | 0,16996293 |
|  |  | hsa-miR-433-3p | 0,17044273 |
|  |  | hsa-miR-106a-5p | 0,17052652 |
|  |  | hsa-miR-320a | 0,17141164 |
|  |  | hsa-miR-141-3p | 0,17419076 |
|  |  | hsa-miR-223-3p | 0,17424691 |
|  |  | hsa-miR-132-3p | 0,17477176 |
|  |  | hsa-miR-15b-3p | 0,17638662 |
|  |  | hsa-miR-30e-3p | 0,1770828 |
|  |  | hsa-miR-1181 | 0,17963321 |
|  |  | hsa-miR-29c-3p | 0,18069055 |
|  |  | hsa-miR-362-5p | 0,18233457 |
|  |  | hsa-miR-663a | 0,18439993 |
|  |  | hsa-miR-376c-3p | 0,18949255 |
|  |  | hsa-miR-451a | 0,19871904 |
|  |  | hsa-miR-636 | 0,19996938 |
|  |  | hsa-let-7i-5p | 0,20016241 |
|  |  | hsa-miR-153-3p | 0,20309421 |
|  |  | hsa-miR-181b-5p | 0,20318531 |
|  |  | hsa-miR-28-3p | 0,20384126 |
|  |  | hsa-miR-598-3p | 0,20510478 |
|  |  | hsa-miR-194-3p | 0,21023649 |
|  |  | hsa-miR-142-5p | 0,21090599 |
|  |  | hsa-miR-18a-3p | 0,21091579 |
|  |  | hsa-miR-595 | 0,21097544 |
|  |  | hsa-miR-301b | 0,21311677 |
|  |  | hsa-miR-760 | 0,2165728 |
|  |  | hsa-miR-23b-3p | 0,21996471 |
|  |  | hsa-miR-590-3p | 0,22313473 |
|  |  | hsa-miR-421 | 0,22638637 |
|  |  | hsa-miR-500a-5p | 0,22651586 |
|  |  | hsa-miR-151a-5p | 0,22864638 |
|  |  | hsa-miR-362-3p | 0,2299205 |
|  |  | hsa-miR-143-3p | 0,23333053 |
|  |  | hsa-miR-133b | 0,23369504 |
|  |  | hsa-miR-425-3p | 0,23460587 |
|  |  | hsa-miR-502-3p | 0,23604045 |
|  |  | hsa-miR-769-5p | 0,23758703 |
|  |  | hsa-miR-18a-5p | 0,23883812 |
|  |  | hsa-miR-423-3p | 0,23976334 |
|  |  | hsa-miR-21-5p | 0,24018184 |
|  |  | hsa-miR-877-5p | 0,24025327 |
|  |  | hsa-miR-424-5p | 0,24564453 |
|  |  | hsa-miR-185-5p | 0,24574109 |
|  |  | mmu-miR-378a-3p | 0,2465416 |
|  |  | hsa-miR-130b-5p | 0,24717025 |
|  |  | hsa-miR-19a-3p | 0,24769214 |
|  |  | hsa-miR-425-5p | 0,24972722 |
|  |  | hsa-miR-128-3p | 0,2506128 |
|  |  | hsa-miR-548d-5p | 0,25082882 |
|  |  | hsa-miR-106b-3p | 0,25372736 |
|  |  | hsa-miR-449a | 0,25405029 |
|  |  | hsa-miR-33a-3p | 0,25832389 |
|  |  | hsa-miR-766-3p | 0,26111731 |
|  |  | hsa-miR-30e-5p | 0,26424608 |
|  |  | hsa-miR-486-5p | 0,26427796 |
|  |  | hsa-miR-326 | 0,26557054 |
|  |  | hsa-miR-660-5p | 0,26624162 |
|  |  | hsa-miR-378a-5p | 0,26835971 |
|  |  | hsa-miR-148a-3p | 0,26886018 |
|  |  | hsa-miR-181a-3p | 0,27167712 |
|  |  | hsa-miR-491-5p | 0,27282089 |
|  |  | hsa-miR-671-3p | 0,27341949 |
|  |  | hsa-miR-937-3p | 0,27362956 |
|  |  | hsa-miR-346 | 0,27753488 |
|  |  | hsa-miR-31-5p | 0,27960615 |
|  |  | hsa-miR-23a-3p | 0,28534318 |
|  |  | hsa-miR-661 | 0,28644618 |
|  |  | hsa-miR-219a-5p | 0,28898249 |
|  |  | hsa-miR-196b-3p | 0,28933946 |
|  |  | hsa-miR-92a-1-5p | 0,2898676 |
|  |  | hsa-miR-140-5p | 0,29007422 |
|  |  | hsa-miR-152-3p | 0,29206249 |
|  |  | hsa-miR-196a-5p | 0,29282487 |
|  |  | hsa-miR-27b-3p | 0,29965783 |
|  |  | hsa-miR-19b-1-5p | 0,30441408 |
|  |  | hsa-miR-484 | 0,30978238 |
|  |  | hsa-miR-93-5p | 0,31604349 |
|  |  | hsa-miR-99a-5p | 0,31786719 |
|  |  | hsa-miR-345-5p | 0,32316033 |
|  |  | hsa-miR-16-5p | 0,32432542 |
|  |  | hsa-miR-324-5p | 0,3247848 |
|  |  | hsa-miR-27a-3p | 0,32688453 |
|  |  | hsa-miR-301a-3p | 0,32845554 |
|  |  | hsa-miR-92a-3p | 0,32876235 |
|  |  | hsa-miR-191-5p | 0,33441837 |
|  |  | hsa-miR-769-3p | 0,33540451 |
|  |  | hsa-miR-186-5p | 0,33784561 |
|  |  | hsa-miR-27a-5p | 0,34204594 |
|  |  | hsa-miR-320d | 0,34320774 |
|  |  | hsa-miR-1244 | 0,34830908 |
|  |  | hsa-miR-361-5p | 0,36182324 |
|  |  | hsa-miR-33b-3p | 0,36461707 |
|  |  | hsa-miR-101-3p | 0,36708473 |
|  |  | hsa-miR-34a-3p | 0,36774305 |
|  |  | hsa-miR-9-3p | 0,36814372 |
|  |  | hsa-miR-24-3p | 0,37219279 |
|  |  | hsa-miR-140-3p | 0,37616721 |
|  |  | hsa-miR-625-3p | 0,37953294 |
|  |  | hsa-miR-505-3p | 0,38079937 |
|  |  | hsa-miR-632 | 0,39761387 |
|  |  | hsa-miR-199a-3p | 0,40236285 |
|  |  | hsa-miR-584-5p | 0,40984832 |
|  |  | hsa-miR-30d-5p | 0,41759374 |
|  |  | hsa-miR-615-3p | 0,43256235 |
|  |  | hsa-miR-125b-5p | 0,43863809 |
|  |  | hsa-miR-582-5p | 0,44100808 |
|  |  | hsa-miR-7-1-3p | 0,44393458 |
|  |  | hsa-miR-423-5p | 0,44416635 |
|  |  | hsa-miR-221-3p | 0,44622616 |
|  |  | hsa-miR-339-3p | 0,45669535 |
|  |  | hsa-miR-210-3p | 0,48741515 |
|  |  | hsa-miR-25-3p | 0,48964498 |
| Increased in EV SAHA |  | hsa-miR-1913 | 2,03641275 |
|  |  | hsa-miR-582-3p | 2,07335621 |
|  |  | hsa-miR-133a-3p | 2,11520574 |
|  |  | hsa-miR-548a-3p | 2,13245752 |
|  |  | hsa-miR-1256 | 2,20567006 |
|  |  | hsa-miR-1537-3p | 2,20601102 |
|  |  | hsa-miR-99b-3p | 2,25878037 |
|  |  | hsa-miR-32-3p | 2,27510953 |
|  |  | hsa-miR-940 | 2,31474617 |
|  |  | hsa-miR-1179 | 2,56437989 |
|  |  | hsa-miR-26a-2-3p | 2,59878089 |
|  |  | hsa-miR-335-3p | 2,68129694 |
|  |  | hsa-miR-483-3p | 3,59946627 |
|  |  | hsa-miR-342-3p | 3,67010159 |
|  |  | hsa-miR-761 | 4,53254392 |
|  |  | hsa-miR-577 | 4,57177372 |
|  |  | hsa-miR-624-5p | 6,63702634 |
|  |  | hsa-miR-188-5p | 7,34937721 |
|  |  | hsa-miR-19b-3p | 73,5024788 |
|  |  | hsa-miR-330-3p | 460994,496 |
