# Supplementary figures and images for: Vorinostat impairs the cancer-driving potential of leukemia-secreted extracellular vesicles
Source: J Transl Med. 2025 Apr 10;23:421. doi: 10.1186/s12967-025-06361-1 (PMC11987450; doi:10.1186/s12967-025-06361-1)

**A**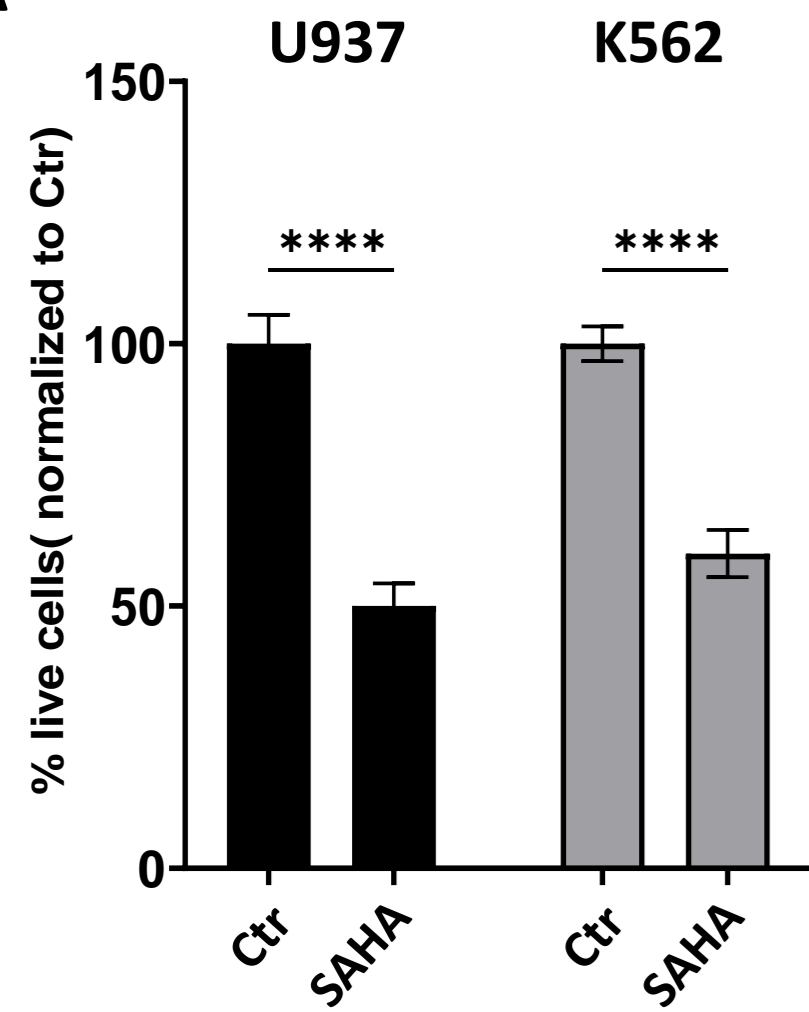**B**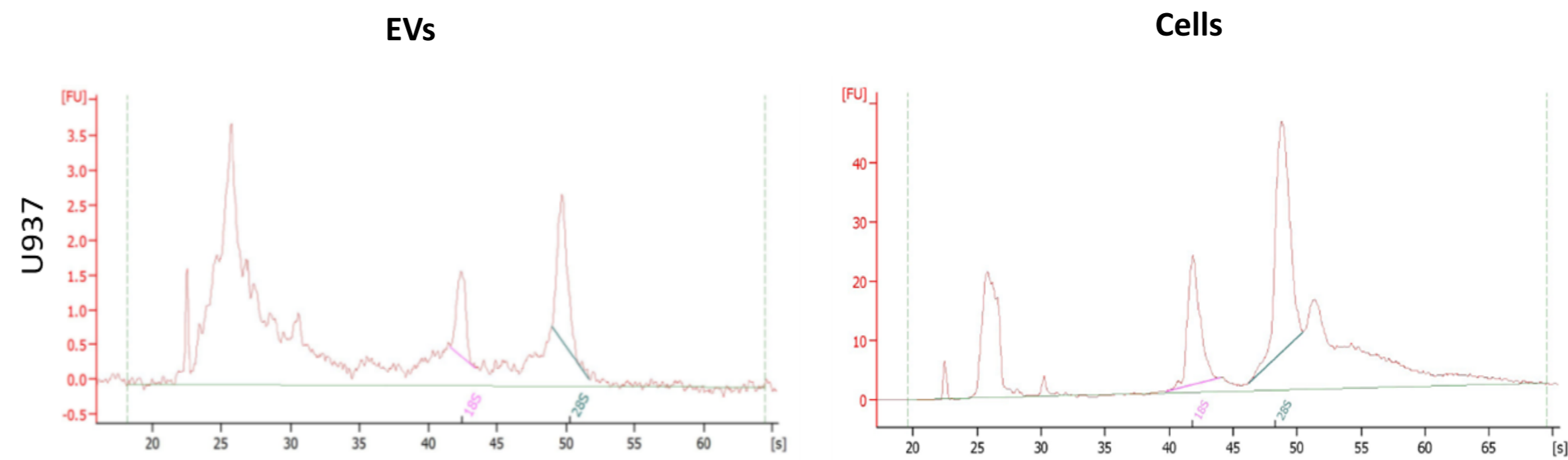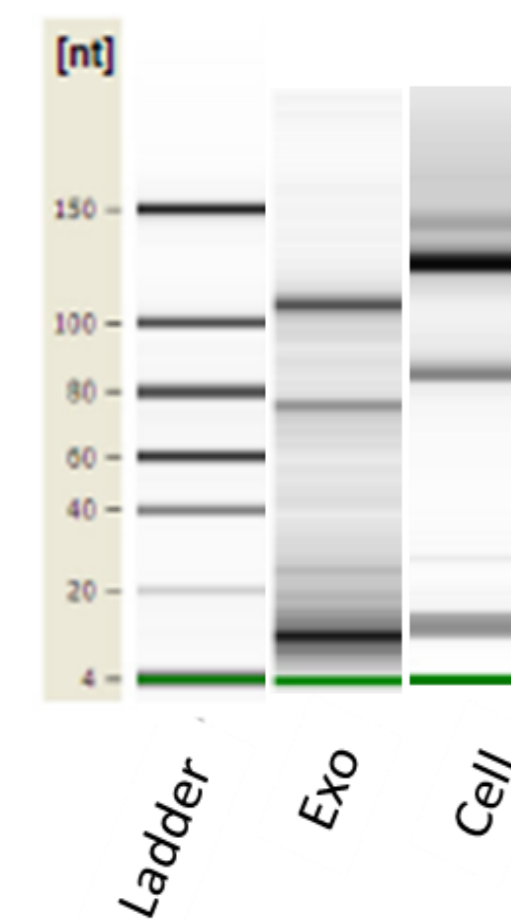**C**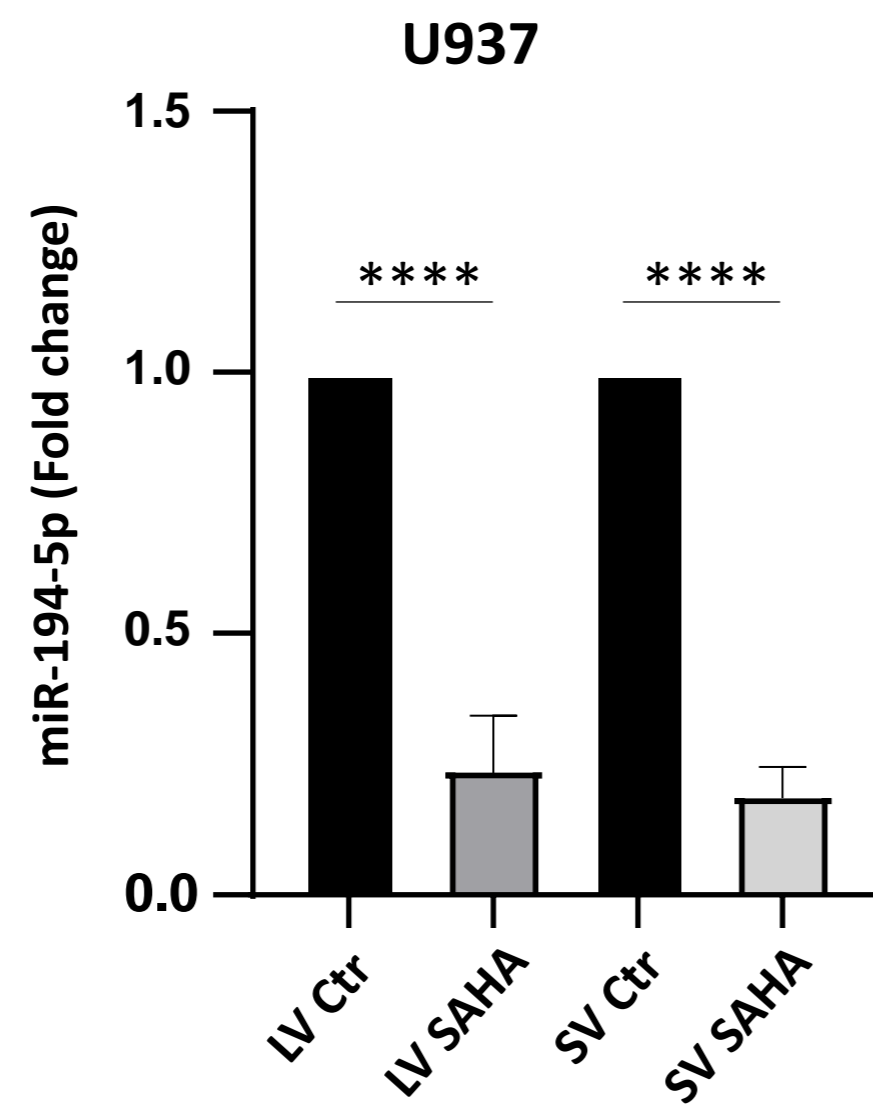**D**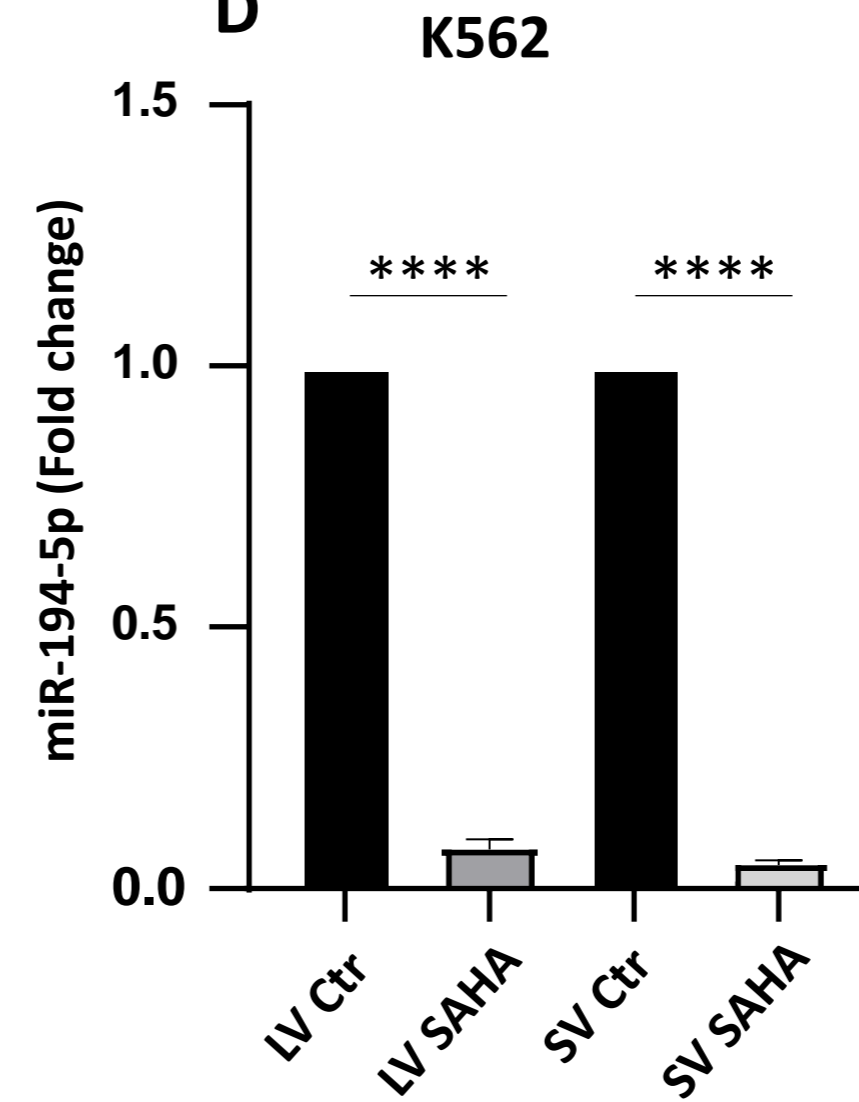

Supplement: Supplementary file 2 — Additional file 2 [file 12967_2025_6361_MOESM2_ESM.pdf]

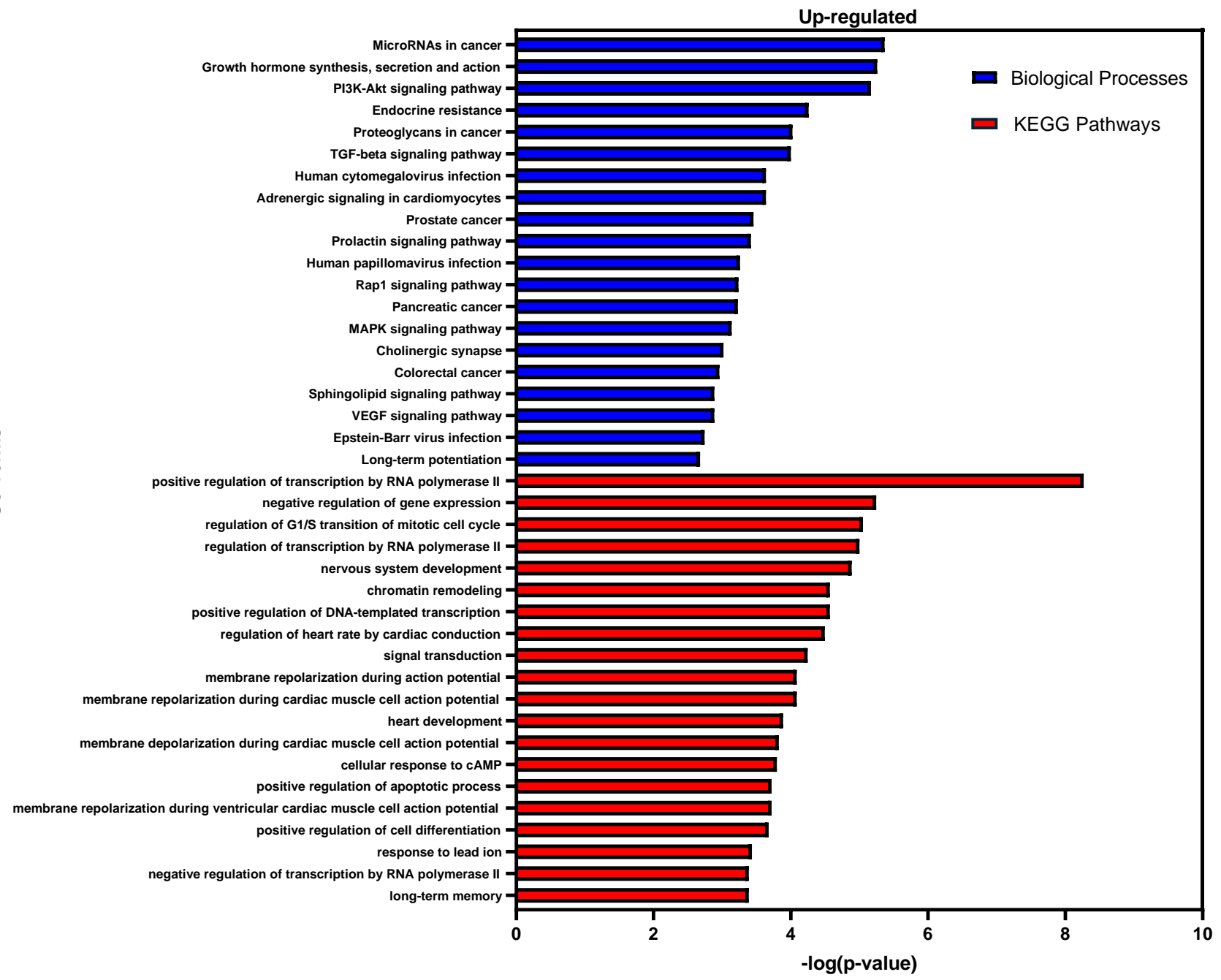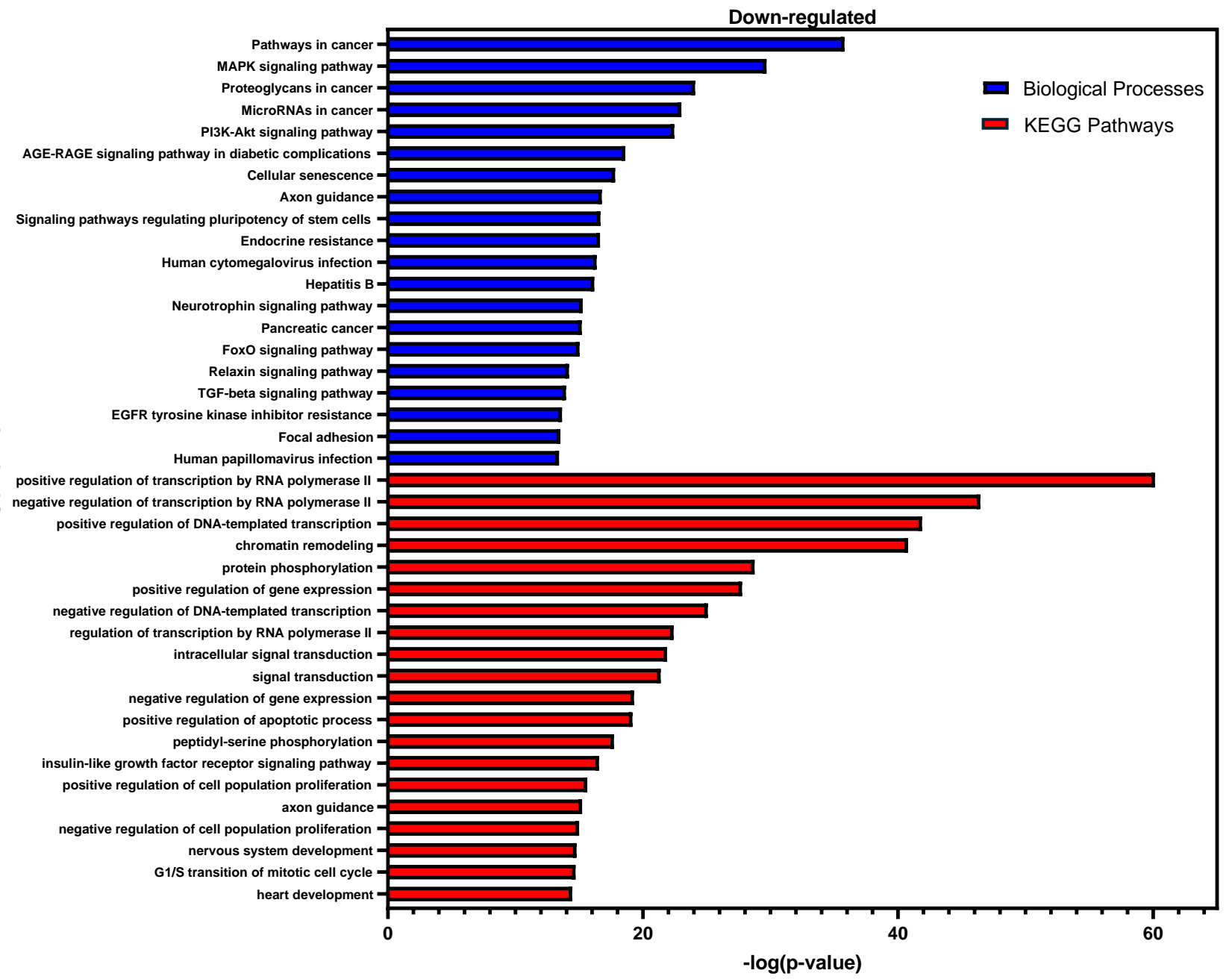

Supplement: Supplementary file 3 — Additional file 3 [file 12967_2025_6361_MOESM3_ESM.pdf]

**A**

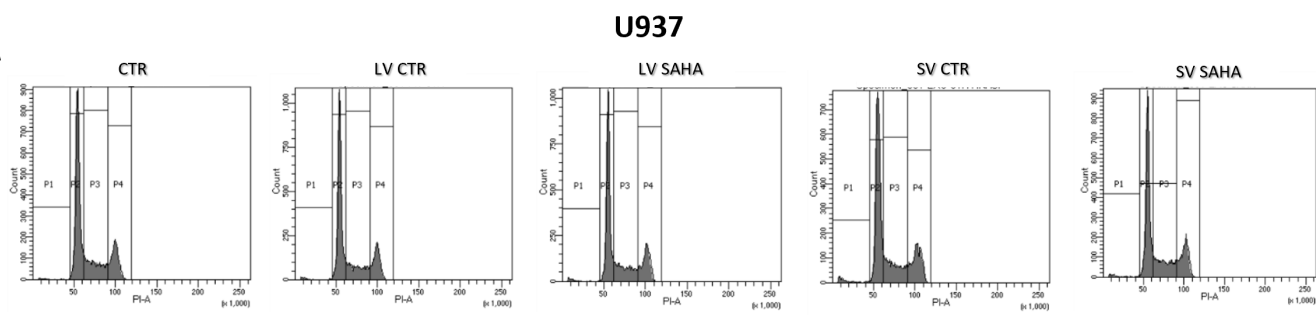

**B**

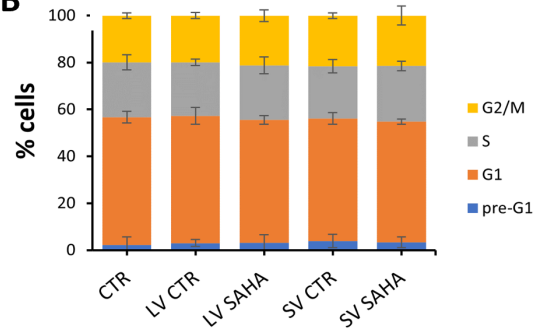

**C**

**K562**

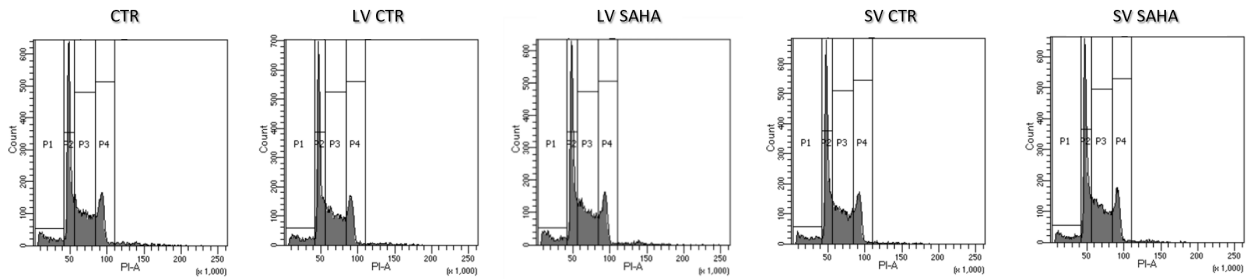

**D**

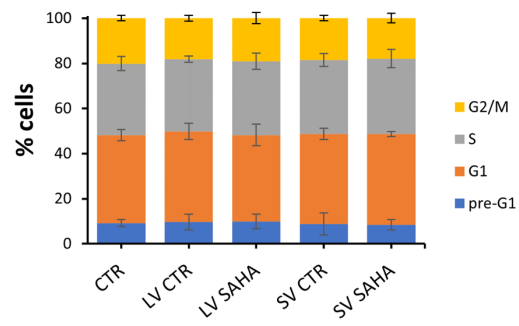

Supplement: Supplementary file 4 — Additional file 4 [file 12967_2025_6361_MOESM4_ESM.pdf]
